# Understanding Myocardial Infarction with Non-Obstructive Coronary Arteries (MINOCA): a comprehensive meta-analysis of clinical characteristics, management, and prognosis compared to MI with the Obstructive Coronary Artery (MIOCA)

**DOI:** 10.1186/s12872-025-04504-2

**Published:** 2025-03-01

**Authors:** Nahid Khorasani, Yaser Mohammadi, Mahdiye Sarpoli, Toba Kazemi, Seyed Mohammad Riahi

**Affiliations:** 1 Student Research Committee, Birjand University of Medical Sciences, Birjand, 9717853577, Iran; 2 Department of Biochemistry, School of Medicine, Iran University of Medical Sciences, Tehran, Iran; 3 Department of Community Medicine, School of Medicine, Cardiovascular Diseases Research Center, Birjand University of Medical Sciences, Birjand, Iran

**Keywords:** Myocardial infarction, MI non-obstructive coronary arteries, MI with the obstructive coronary artery, Acute Coronary Disease, MINOCA, MIOCA

## Abstract

**Background:**

MINOCA (Myocardial Infarction with Non-Obstructive Coronary Arteries) represents a unique subset of acute coronary syndrome, distinct from MIOCA (Myocardial Infarction with Obstructive Coronary Arteries) and a control group. This study systematically compares their prevalence, clinical characteristics, management strategies, and outcomes to improve understanding and treatment approaches.

**Methods:**

This systematic review and meta-analysis followed PRISMA guidelines across multiple databases up to 2024. STATA 17 was used for statistical analyses, and the Newcastle-Ottawa Scale was employed to assess study quality.

**Results:**

One-hundred and twelve studies, including 5,908,768 patients, were analyzed. The pooled prevalence of MINOCA among patients undergoing coronary angiography was 8.92% (95% CI: 8.90–8.94). MINOCA patients were generally younger, predominantly female, and more likely to present with atypical chest pain and dyspnea compared to MIOCA patients. Laboratory findings showed higher levels of CRP, BNP, and fibrinogen in MINOCA patients, suggesting inflammation and microvascular dysfunction as key mechanisms. In contrast, MIOCA patients had higher rates of diabetes and dyslipidemia, highlighting differences in pathophysiological processes. Medication use differed between the groups, with MINOCA patients more likely to be prescribed anticoagulants and β-blockers. Prognostically, MINOCA patients experienced significantly lower rates of adverse short- and long-term outcomes, including major adverse cardiac events (MACE) and cardiovascular death, compared to MIOCA patients.

**Conclusions:**

This study demonstrated that patients with MINOCA have a better prognosis compared to those with MIOCA and are at a lower risk of serious cardiac events. Based on the findings of this study, we emphasize that microcirculation and vascular spasm are the main mechanisms involved in MINOCA. Considering these findings, it is suggested that a better management strategy for MINOCA patients can be established by precisely defining diagnostic criteria and focusing on anti-inflammatory treatments and risk factor control.

## Introduction

Acute myocardial infarction (MI) is recognized as one of the most common cardiovascular diseases and is considered one of the leading causes of mortality and disability worldwide each year [1]. MI was traditionally defined as a complication of coronary artery disease (CAD) resulting from coronary artery obstruction [2]. However, with advancements in diagnostic methods, it has been observed that despite the occurrence of MI symptoms, significant obstruction is not always present [3]. This condition is now known as myocardial infarction with non-obstructive coronary arteries (MINOCA) [4].

MINOCA is a clinical syndrome characterized by clinical evidence of myocardial infarction with normal or near-normal coronary arteries on angiography [5]. This condition is observed in approximately 5–7% of patients [6]. Due to the wide range of underlying mechanisms, MINOCA is recognized as a heterogeneous condition [6, 7]. These mechanisms may include coronary causes such as eccentric plaques, coronary artery rupture, vascular spasm, microvascular embolism, and rapidly resolved thrombosis [8, 9]. Additionally, non-coronary causes such as Takotsubo cardiomyopathy, myocarditis, microvascular spasm, and myocardial oxygen supply-demand imbalance may also play a role [10, 11]. This diversity in underlying mechanisms makes the accurate diagnosis and proper management of MINOCA a clinical challenge.

From a clinical perspective, significant differences exist between MINOCA patients and those with obstructive coronary artery disease (MIOCA). Studies have shown that MINOCA patients are generally younger, more frequently observed in women, and exhibit distinct electrocardiographic patterns [12, 13]. Furthermore, MINOCA is associated with poor prognosis, and if not accurately diagnosed and properly treated, it can lead to serious cardiovascular complications.

Given the specific complexities associated with MINOCA, its medical treatment remains uncertain [14]. Due to the lack of sufficient information and the absence of specific treatment protocols, physicians often resort to treatment patterns similar to those used for classical myocardial infarctions [15]. Common treatments include the use of β-blockers, antiplatelet agents, statins, and ACE Inhibitors (ACE-I); however, their effectiveness is not yet fully understood [16, 17]. Consequently, the absence of clear guidelines for drug prescriptions presents a challenge for physicians, highlighting the need for further studies.

To our knowledge, no comprehensive systematic review and meta-analysis on MINOCA has been conducted to date. Given the clinical and diagnostic heterogeneity and complexity of MINOCA, as well as the lack of specific treatment protocols, this study aims to examine the demographic, clinical, and laboratory characteristics of MINOCA patients and compare them with MIOCA patients and healthy individuals. Additionally, this research seeks to assess the short- and long-term prognosis of MINOCA patients compared to MIOCA and control groups and aims to identify clinical differences and secondary preventive treatments to improve the management of these patients.

## Methods

### Protocol registration

The methodology of this study followed the guidelines outlined in the Preferred Reporting Items for Systematic Reviews and Meta-analyses (PRISMA) [18]. Ethical approval for the study was obtained from Prospero (BUMS ID: CRD42023442971) and Birjand University of Medical Sciences (ID: IR.BUMS.REC.1401.448).

### Search strategy

Table 1 outlines the search strategy and the associated keywords used in this study. Two independent researchers (M.Y. and SP.M.) conducted comprehensive searches across the SCOPUS, EMBASE, PubMed, and Web of Science databases up until July 2024, adhering to PRISMA guidelines. The search utilized a combination of both MeSH and non-MeSH terms, employing Boolean operators (AND, OR) to refine the strategy. There were no geographical or language restrictions imposed during the search process. To ensure a thorough investigation, we also explored databases and gray literature to identify any potentially overlooked studies. Additionally, an automated search was performed within Google Scholar to capture the latest available publications in the field.

**Table 1:** Search strategy

Query	Search Strategy	PubMed	Scopus	Web of Science	Embase
#1	“acute coronary syndrome” OR “acute myocardial infarction” OR “arteriosclerosis” OR MI OR CAD OR CVD OR “cardiovascular disease” OR “cardiovascular events” OR “coronary heart disease” OR “coronary disease” OR “coronary occlusion” OR “coronary thrombosis” OR “coronary stenosis” OR “ischemic heart disease” OR “heart attack” OR “heart disease” OR “atherosclerotic” OR “myocardial infarction” OR “coronary artery disease”	817804	1795426	344143	230834
#2	“nonobstructive coronary” OR “non obstructive coronary” OR “non obstructed coronary” OR “no obstructive coronary” OR “without obstructive coronary” OR “without obstructed coronary” OR ‘MINOCA OR “myocardial infarction with nonobstructive coronary arteries”	2117	2752	1745	991
#3	#1 AND #2	1932	2672	1370	562

This table shows the search strategy, including key terms, databases (PubMed, Scopus, Web of Science, Embase), and the number of results retrieved

### Study selection (inclusion & exclusion criteria)

MINOCA was defined in this meta-analysis according to the universal definition proposed by the European Society of Cardiology (ESC) [19]: myocardial infarction with non-obstructive coronary arteries (stenosis < 50%) and the absence of alternate clinically overt diagnoses, such as myocarditis or Takotsubo cardiomyopathy, based on clinical, laboratory, and imaging criteria. Studies that used a different definition of obstruction (e.g., stenosis ≥ 50%) or did not provide sufficient data to exclude alternative diagnoses were excluded from the analysis.

The control group included patients who underwent coronary angiography due to symptoms suggestive of coronary artery disease (CAD), such as stable or unstable angina. Despite presenting with clinical indications of CAD, these patients demonstrated no evidence of coronary artery obstruction on angiography. This contrasts with MINOCA patients, who exhibit partial coronary obstruction (< 50%) on angiography. The inclusion of this control group provides a valuable comparison for understanding the unique characteristics of MINOCA (ESC) [20].

**Fig. 1:**
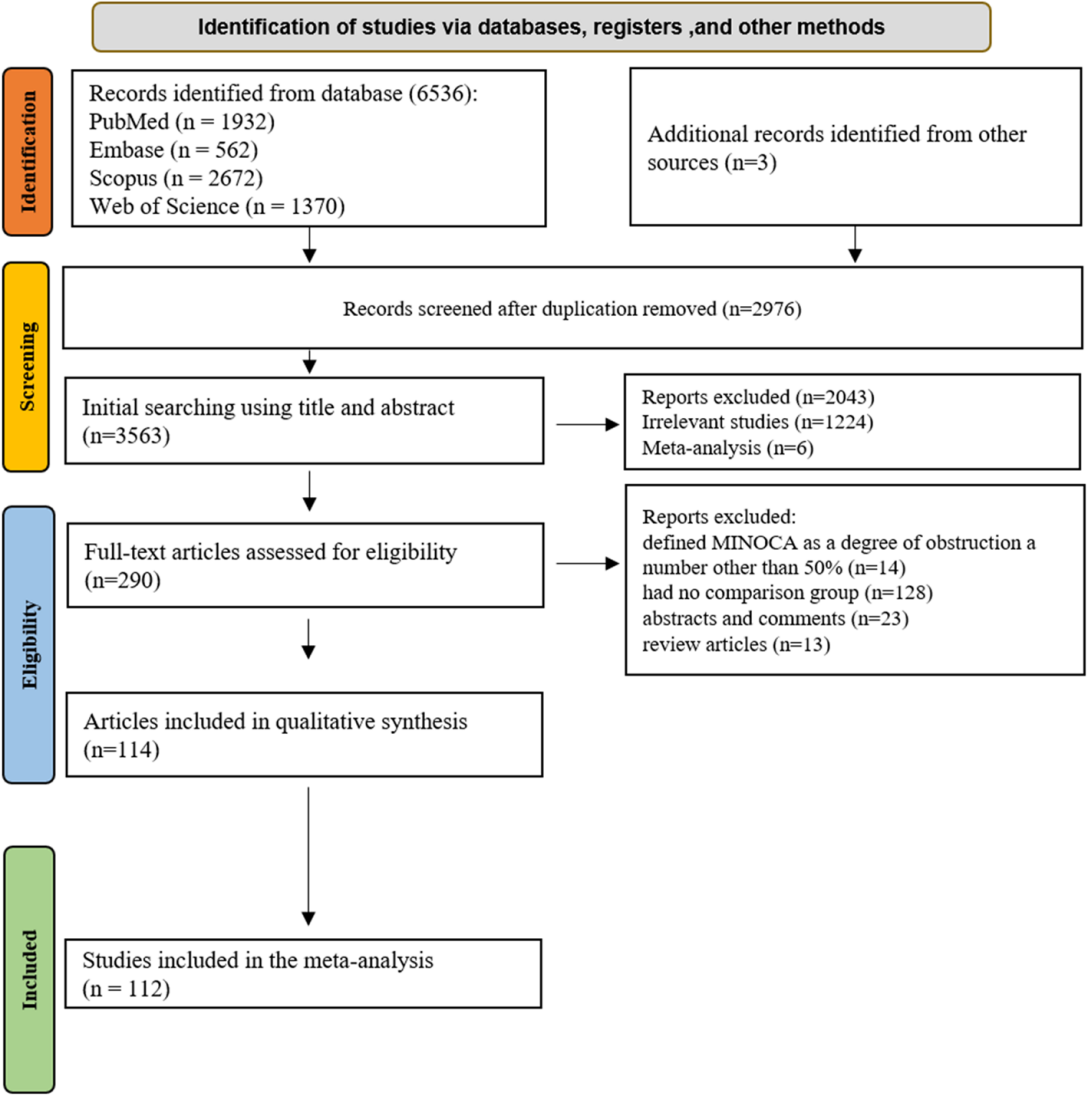
Study selection flowchart. Displays the PRISMA flowchart showing the number of studies identified, screened, excluded, and included in the meta-analysis

After importing the search results from databases into EndNote X9, duplicate entries were identified and removed using the “Find Duplicates” tool. The screening process was conducted in two stages by two independent reviewers (KH.N. and SP.M.). The first stage involved screening based on titles and abstracts, while the second stage included a detailed review of the full-text studies. Any disagreements were resolved through direct consultation or by involving a third reviewer (R.S.M.). Inclusion criteria focused on original observational studies examining MINOCA patients and their comparison with the MIOCA control group, or both. Exclusion criteria were applied to studies that: (1) used a different definition of obstruction for MINOCA other than 50%, (2) lacked a comparison group, (3) were reviews, conference abstracts, protocols, editorials, case reports, letters, or commentaries, (4) involved animal studies, or (5) had incomplete data. The selection process is summarized in Fig. 1.

### PECOS framework

The research question was formulated using the PECOS framework, ensuring a structured and comprehensive approach to the study. Two key comparisons were explored: first, between patients with MINOCA and those with MIOCA, and second, between MINOCA patients and a control group who had no evidence of CAD on angiography.

- ▪ P (Population): patients who underwent coronary angiography.
- ▪ E (Exposure): MINOCA.
- ▪ C (Control/ Comparison): MIOCA or control (no angiographic CAD).
- ▪ (Outcome): short-term (in-hospital) and long-term (one-year follow-up) prognosis.
- ▪ S (Study design): cohort, cross-sectional, and case-control observational studies.

### Data extraction

Using Excel software, a data extraction form was created to gather data ffrom the studies included in the meta-analysis. These details included author, country, publication year, study design, patients’ demographic and medical history, symptoms and test results at the time of admission, medication prescription at the onset, and discharge and outcomes, Table 1 has further information. The final data was obtained after these extracted data, which were collected independently by two authors (SP.M. and KH.N.), were compared to identify and confirm any differences.

### Risk of bias assessment

For studies that satisfied the eligibility requirements, the validated Newcastle-Ottawa Scale (NOS) tool was used to assess the risk of bias [21]. This instrument assesses research from three angles: the choice of study groups, group comparability, and evaluation of the desired outcome. There is one point given to each of these requirements. A study can be given a number between 0 and 9, where higher numbers denote greater quality and lower chance of bias. Studies with a score of less than 4 often have a higher risk of bias, while those with a score of 7 to 9 are considered high-quality and have the least possibility of bias. Three reviewers assessed the quality of the studies on an individual basis (M.Y., SP.M., and K.T.). The reviewers discussed any disagreements regarding the grading and, if necessary, spoke with the fourth reviewer (R.S.M). Because there was a significant probability of bias overall, studies having a total score of less than 6 on the NOS instrument were not included in the final analysis.

### Statistical analysis

Metaprop was used to estimate the pooled prevalence of MINOCA in the population undergoing coronary angiography using cross-sectional studies (*n* = 69) that reported the total number of angiographic populations. The pooled prevalence was calculated using a random-effects model to account for variability across studies. Additionally, pooled prevalence for each variable in each study group was calculated. The result of comparisons between study groups was expressed in terms of odds ratio (OR) and risk ratio (RR) for binary data, and standardized mean difference (SMD) for continuous data.

In the articles that reported the first and third quartiles, we used the following to convert them into mean values [22, 23]; Mean = (IQR (q3 - q1)) / 1.35. This approach was employed to harmonize the format of the reported data across studies, enabling a consistent meta-analysis. The use of formulas based on the normal distribution assumption assumes symmetric data, which may not always hold true in meta-analyses where skewed distributions are common. Also, it is important to note that this method does not provide an estimate for standard deviation (SD) but is primarily used to approximate the mean from IQR. Despite these limitations, these methods offer a practical approach when raw data are unavailable [24].

To obtain the complications after CAD, we used the cohort articles (*n* = 81). To equalize the duration of follow-ups to perform the analysis in terms of one-year follow-up, we multiplied the percentage of complications by 12 (12 months) and divided by the number of months of the follow-up period of each study. The use of this approach has certain limitations. First, it assumes a symmetric distribution of the data, which may not always be true. Second, it introduces variability when comparing studies with different reporting methods. Despite these limitations, this method provides a practical solution for standardizing data in the absence of raw individual-level data.

Heterogeneity between studies was quantified using the I^2^ test and chi-square (Cochran Q) test, considering I^2^ ˃70% as statistically heterogeneous [25, 26]. Random-effects and meta-regression analyses were performed to evaluate whether the differences in the follow-up duration of the studies contributed to heterogeneity [27]. Additionally, publication bias was assessed using Egger’s tests, and funnel plots were generated for key outcomes to visually evaluate the presence of bias. Sensitivity analysis was performed based on different study designs and different follow-ups to validate the robustness of the analyses. All statistical analyses were carried out using STATA version 17 (STATA Corp., College Station, Texas). *P*-values less than 0.05 were regarded as statistically significant.

## Results

### Search results

Through an electronic search of the databases, a total of 6,536 articles were identified: 1,932 from PubMed, 562 from EMBASE, 2,672 from SCOPUS, and 1,370 from Web of Science. After importing the search results into EndNote software, 2,976 studies were removed using the “Find Duplicates” tool. In the first screening phase, the titles and abstracts were reviewed, and 3,273 articles that did not meet the inclusion criteria were excluded. In the second phase, the full texts of 290 articles were thoroughly examined, with 176 being excluded for failing to meet the eligibility criteria. Additionally, to ensure no studies were overlooked, gray literature was also reviewed. A total of 112 eligible studies were included in the meta-analysis. Figure 1 provides a detailed overview of the article selection process.

### Quality assessment

**Fig. 2:**
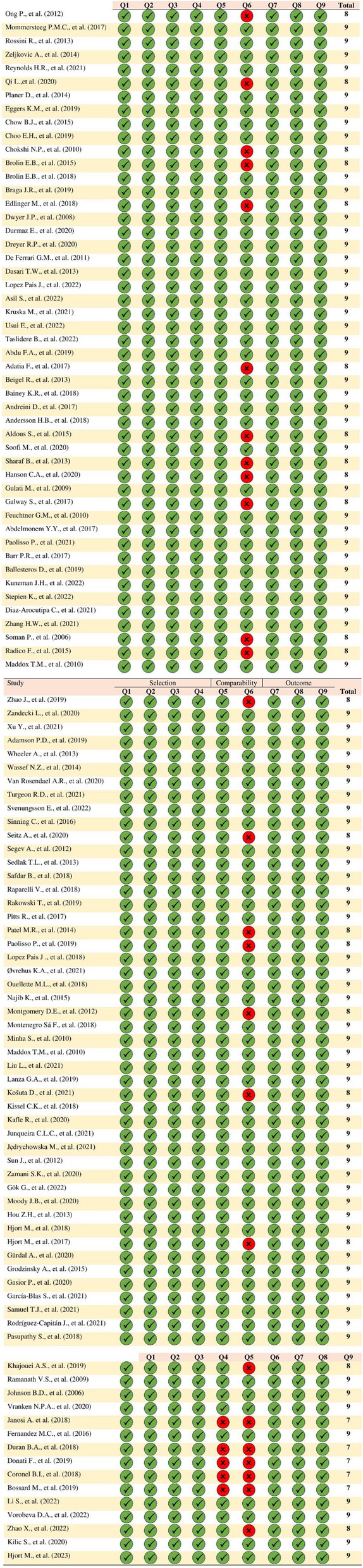
Quality assessment of included articles Illustrates the Newcastle-Ottawa Scale evaluation of the methodological quality of included studies. Selection: Q1- Was the case definition adequate? Q2- Were the cases representative? Q3- Were the controls selected? Q4- Were the controls clearly defined? Comparability: Q5- Was the adjustment made for the most important risk factors? Q6-Was the adjustment made for other risk factors? Outcome: Q7- Was the outcome assessment adequate? Q8- Was the follow-up length sufficient? Q9- Was the follow-up process adequate? Each plot represents the publication bias assessment for the corresponding outcome. The symmetrical distribution around the pooled effect size suggests no significant bias, as confirmed by Egger’s tests (*p*-values > 0.05)

Figure 2 provides a detailed depiction of the quality assessment conducted for the included studies. Each eligible study was evaluated using the NOS tool. Based on this evaluation, the results indicate a range of quality scores. Specifically, five studies received a score of 7, reflecting a medium to high level of quality. twenty-one studies were rated with a score of 8, indicating good quality. The majority of the studies, totaling eighty-six papers, achieved a score of 9, demonstrating a high level of methodological rigor and robustness. These scores suggest that the overall quality of the studies included in this review is satisfactory, with a significant proportion of high-quality research. This thorough assessment process ensures the reliability and validity of the findings presented in this systematic review and meta-analysis.

### Publication bias

We assessed publication bias using the Egger tests. The results showed no evidence of significant publication bias affecting the findings. The *p*-values for the Egger test are presented in Tables 2, 3, 4, and 5.

**Table 2:** Characteristics of included studies

First Author (Year)	Country	WHO Region	Study Design	Sample Size in Study Groups				Follow-up (Days)	Investigated Variables in Each Study				NOS Score (%)
				**Total Sample**	**MINOCA**	**MIOCA**	**Control**		**Medical History**	**Symptoms/ Laboratory Data at Admission**	**Treatment at Admission/ Discharge**	**Outcomes**	
Zhao J., et al. (2019) [28]	China	Western Pacific	Cross-Sectional	345	129	133	83	NA	HTN, DM, Smoke, HLP	NA	NA	NA	8
Zandecki L., et al. (2020) [29]	Poland	European	Cohort	9771	2120	7624	NA	543.5 ± 451.85	DM, Stroke, MI, PCI, CABG, Smoke, HTN, Kidney Disease, COPD,	NA	NA	MACE, Death, MI, Stroke, HF	9
Xu Y., et al. (2021) [30]	China	Western Pacific	Cross-Sectional	503	20	156	138	NA	DM, HTN, DLP, Smoke	NA	NA	NA	9
Adamson P.D., et al. (2019) [31]	New Zealand	Western Pacific	Cohort	8305	897	7408	NA	412.45 ± 219	Smoke, DM, Dementia, Liver Disease, Cancer, CVD	SBP/ HDL, LDL	NA/ Statin, Bata-Blocker, CCB, Aspirin, ACE-I or ARB, Clopidogrel	Death, CVA, MI	9
Wheeler A., et al. (2013) [32]	Australia	Western Pacific	Cross-Sectional	4312	325	819	3168	NA	HTN, DM, Smoke, HLP, MI, Angina, HF, Stroke, TIA	NA	NA	NA	9
Wassef N.Z., et al. (2014) [33]	UK	European	Retro-Cohort	1351	115	847	NA	365	HTN, DM, Smoke, DLP	NA/ Hb, Cr, GFR	NA	Death, CV-Death, CVA, Revascularization, Bleeding	9
Van Rosendael A.R., et al. (2020) [34]	USA	Americas	Pro-Cohort	6620	1698	NA	1849	1825	HTN, DM, HLP, Smoke	CP, Dyspnea/ NA	Statin, Beta-Blocker, Aspirin, ACE-I/ NA	NA	9
Turgeon R.D., et al. (2021) [35]	Canada	Americas	Cohort	4996	2132	444	1707	839.5	HTN, DM, DLP, Smoke, Obesity, CVD, PAD, HF	CP, Dyspnea, SBP/ GFR, LDL	Statin, Beta-Blocker, Anti-Thrombotic, RAAS-I/ Statin, Beta-Blocker	NA	9
Svenungsson E., et al. (2022) [36]	Sweden	European	Case–Control	297	98	99	9	NA	HTN, DM, HLP, Smoke	NA/ Hb, Cr, GFR, Platelets, CRP, BNP, TC, TG, LDL, HDL	NA	NA	9
Sinning C., et al. (2016) [37]	Germany	European	Cohort	2316	350	1966	NA	1789	HTN, DM, DLP, Smoke, MI	NA/ Cr, LDL, HDL	Statin, Beta-Blocker, ACE-I/ NA	CV-Death, MI	9
Seitz A., et al. (2020) [38]	Germany	European	Pro-Cohort	376	103	109	NA	1825	MI	NA	NA	Death, CV-Death, MI	8
Segev A., et al. (2012) [39]	Israel	European	Cohort	444	115	NA	266	912.5 ± 146	HTN, DM, DLP, Smoke	NA/ CRP, LDL, HDL, TG	NA	MACE, Death, Revascularization	9
Sedlak T.L., et al. (2013) [40]	Canada	Americas	Cohort	13695	98	10608	2079	694	HTN, DM, MI, PCI, CABG, Kidney Disease, PVD, CVA, HF, COPD, Liver Disease, Cancer, Smoke	NA	NA	NA	9
Safdar B., et al. (2018) [41]	Italy	European	Pro-Cohort	2985	299	2374	NA	365	HTN, DM, DLP, Smoke, Obesity, Stroke, TIA, MI, HF, PAD, Depression, Kidney Disease, PCI, CABG	NA	NA/ Statin, Beta-Blocker, Aspirin, ACE-I or ARB	Death	9
Raparelli V., et al. (2018) [42]	Canada	Americas	Pro-Cohort	1210	82	916	NA	365	Angina, PAD, DM, HTN, Smoke, DLP, ACS, Depression,	SBP, DBP, CP, Dyspnea/ Troponin, CRP	NA/ Statin, Bata-Blocker, CCB, Aspirin, ACE-I, ARB	MACE, CV-Death, MI, Stroke, HF	9
Rakowski T., et al. (2019) [43]	Poland	European	Cohort	49893	3924	45969	NA	NA	DM, Smoke, HTN, Kidney Disease, Stroke, MI, COPD	NA	Aspirin, Heparin, P2Y12-I/ Nitrates	NA	9
Pitts R., et al. (2017) [44]	Colorado	Americas	Cohort	3830	200	3630	NA	365	HTN, DM, Smoke, CAD, MI, CABG, PCI, PVD, Stroke, TIA, DLP, Kidney Disease, Depression	NA	Statin, Beta-Blocker, Aspirin, ACE-I, ARB, Warfarin/ NA	Death	9
Patel M.R., et al. (2014) [45]	New York	Americas	Cross-Sectional	661063	386003	275060	NA	NA	DM, HF	CP/ NA	NA	NA	8
Paolisso P., et al. (2019) [46]	Italy	European	Cohort	1523	134	998	66	580.5 ± 319.5	HTN. DM, Smoke, DLP, MI, Stroke, AF	NA/ GFR, Hb	NA/ Statin, Beta-Blocker, Aspirin, P2Y12-I, RAAS-I	NA	8
Lopez Pais J ., et al. (2018) [47]	Italy	European	Cross-Sectional	387	118	269	NA	NA	DM, DLP	NA/ Troponin-I	NA	NA	9
Øvrehus K.A., et al. (2021) [48]	Denmark	European	Cohort	33552	13883	NA	19669	1277.5	HTN, DM, HLP, Smoke	CP, Dyspnea/ NA	NA	Death, MI	9
Ouellette M.L., et al. (2018) [49]	America	Americas	Pro-Cohort	925	412	513	NA	NA	HTN, DM, HLP, Smoke, PVD, CVD, Kidney Disease	CP/ NA	Statin, Beta-Blocker, Aspirin, ACE-I, ARB/ Statin, Beta-Blocker, Aspirin, ACE-I or ARB	NA	9
Najib K., et al. (2015) [50]	Chicago	Americas	Retro-Cohort	2038	216	1822	NA	365	HTN, DM, HLP, HF, PVD, CVD, MI, PCI, CABG, Kidney Disease	NA	NA	Death, CV-Death	9
Montgomery D.E., et al. (2012) [51]	USA	Americas	Cross-Sectional	146	67	56	NA	NA	HTN, DM, DLP, Smoke	SBP, DBP/ Cr	Statin, Beta-Blocker, Aspirin, CCB, ACE-I, ARB	NA	8
Montenegro Sá F., et al. (2018) [52]	Portugal	European	Retro-Cohort	1047	114	933	NA	1050 ± 21.5	HTN, DM, DLP, Smoke, MI, Stroke, HF, AF, Kidney Disease	NA/ Troponin-I	NA	NA	9
Minha S., et al. (2010) [53]	Israel	European	Cohort	5937	142	2761	NA	365	HTN, DM, DLP, Smoke, CVA, PVD	SBP, DBP/ Cr, GFR, Hb, LDL, HDL, TC, TG	Beta-Blocker, CCB, Aspirin, ACE-I, ARB, Anti-Coagulants, Clopidogrel, Diuretics/ Beta-Blocker, CCB, Aspirin, ACE-I or ARB, Clopidogrel, LMWH	MACE, Death,MI, HF, Cardiogenic Shock, Pulmonary Edema, Pericarditis	9
Maddox T.M., et al. (2010) [54]	America	Americas	Retro-Cohort	2479874	237167	1252578	NA	NA	Obesity, MI, PCI, CABG, HF, HTN, DM, CVD, PVD, Chronic Lung Disease, Smoke, DLP	CP/ NA	Statin, Beta-Blocker, Aspirin, Clopidogrel/ NA	NA	9
Liu L., et al. (2021) [55]	China	Western Pacific	Retro-Cohort	506	232	274	NA	720	Smoke, DM, HTN, HLP	CP/ NA	Statin, Beta-Blocker, CCB, Aspirin, ACE-I, ARB, Clopidogrel/ NA	NA	9
Lanza G.A., et al. (2019) [3]	Italy	European	Retro-Cohort	2163	113	317	NA	NA	Smoke, DLP, HTN, DM, Obesity, AF, PCI	NA/ Cr	NA	NA	9
Košuta D., et al. (2021) [56]	Slovenia	European	Retro-Cohort	1031	284	256	449	1606 ± 27.23	DLP, HTN, DM, Smoke	NA	NA	NA	8
Kissel C.K., et al. (2018) [57]	Canada	Americas	Pro-Cohort	18384	7478	10906	NA	2372.5	HTN, DM, DLP, Smoke	NA	Statin, Beta-Blocker, CCB, Aspirin, ACE-I, Insulin, P2Y12-I/ NA	NA	9
Kafle R., et al. (2020) [58]	Nepal	south-east Asia	Cross-Sectional	177	24	153	NA	NA	Smoke, HTN, DM, DLP, AF	SBP, DBP/ NA	NA	NA	9
Junqueira C.L.C., et al. (2021) [59]	Brazil	Americas	Case-Control	50	25	NA	25	NA	HTN, DLP, Smoke	NA	NA	NA	9
Jędrychowska M., et al. (2021) [60]	Italy	European	Retro-Cohort	1177218	5695	124663	NA	30	DM, HTN, Stroke, MI, PCI, CABG, Smoke, Kidney Disease, COPD	NA	NA/ Aspirin, Clopidogrel, LMWH	Death, Cardia Arrest, Bleeding	9
Sun J., et al. (2012) [61]	China	Western Pacific	Retro-Cohort	736	51	678	NA	1095	HTN, DM, HLP, Smoke, CVD	NA/ Troponin-T, CK-MB, TC, LDL, EF	NA	MACE, Death, CV-Death, MI, Cardiogenic Shock	9
Zamani S.K., et al. (2020) [62]	California	Americas	Cross-Sectional	127	64	NA	39	NA	HTN, DM, DLP, MI	SBP, DBP/ EF	Beta-Blocker, CCB, Nitrates, Diuretics/ NA	NA	9
Gök G., et al. (2022) [63]	Turkey	European	Cross-Sectional	1793	49	428	NA	NA	Smoke, CAD, HTN. HLP, DM	NA/ Troponin-T, Hb, GFR	NA	NA	9
Moody J.B., et al. (2020) [64]	UK	European	Cross-Sectional	231	131	9	NA	NA	Obesity, CAD, HTN, HLP, DM, Smoke, MI, PCI	NA	NA	NA	9
Hou Z.H., et al. (2013) [65]	California	Americas	Cross-Sectional	174	84	NA	90	NA	DM, Smoke, HTN, Kidney Disease, Stroke, MI, COPD	SBP, DBP/ TC	NA	NA	9
Hjort M., et al. (2018) [11]	Sweden	European	Retro-Cohort	18943	1639	17304	NA	365	Smoke, DM, HTN, HLP, HF, Stroke	NA/ GFR, EF	Statin, Beta-Blocker, Aspirin, Anti-Coagolant, P2Y12-I, RAAS-I / Statin, Beta-Blocker, Aspirin, P2Y12-I, RAAS-I	MACE, Death, CV-Death, HF, MI, Stroke	9
Hjort M., et al. (2017) [5]	Sweden	European	Retro-Cohort	19377	1774	16576	1027	330	NA	NA	NA	Death, CV-Death, MI, HF, Stroke	8
Gürdal A., et al. (2020) [66]	Turkey	European	Retro-Cohort	320	72	NA	248	630	Smoke, HTN, DM	NA/ TC, TG, HDL, LDL, Cr, Hb, Platelet, EF	NA	Death	9
Grodzinsky A., et al. (2015) [67]	USA	Americas	Pro-Cohort	5539	381	4941	NA	365	HTN, DM, Smoke, MI	NA/ TC, TG, HDL, LDL, Troponin	NA/ Statin, Beta-Blocker, Aspirin, ACE-I or ARB, Clopidogrel	NA	9
Gasior P., et al. (2020) [68]	Poland	European	Retro-Cohort	205606	6063	160886	NA	1080	HTN, DM, Obesity, Smoke, CAD, PCI, MI, CABG, Stroke, Kidney Disease, Lung Disease, AF	Dyspnea, SBP, DBP/ EF	NA/ Statin, ACE-I, Aspirin, LMWH, Nitrates, P2Y12-I	Death,CV-Death, Cardia Arrest,MI, Stroke, Cardiogenic Shock, Pulmonary Edema,	9
García-Blas S., et al. (2021) [69]	Spain	European	Cohort	591	121	470	NA	1825	HTN, DLP, DM, Smoke, MI, PCI, PAD, HF, Stroke	CP, SBP, DBP/ Hb, Cr, Troponin, GFR	Beta blocker, ACE, Statin/ Clopidogrel, Beta blocker, Statin, ACE-I, Aspirin, Anti-Coagulant	MACE, Death, Revascularization	9
Samuel T.J., et al. (2021) [70]	USA	Americas	Cross-Sectional	77	65	NA	12	NA	HTN, DM, HLP, Smoke	SBP, DBP/ NA	Beta-Blocker, ACE-I, ARB, Statin	NA	9
Rodríguez-Capitán J., et al. (2021) [71]	Spain	European	Retro-Cohort	3265	2069	1196	NA	1290	Smoke, HTN, DM, DLP, HF, Kidney Disease, AF	NA	NA	NA	9
Pasupathy S., et al. (2018) [72]	Australia	Western Pacific	Case-Control	440	25	25	NA	NA	HTN, HLP, DM, Smoke, CAD, PCI	NA	NA/ Beta-Blocker, Statin, ACE-I, ARB, Nitrates, CCB, Anti-Platelet	NA	9
Ong P., et al. (2012) [73]	UK	European	Cross-Sectional	376	144	139	NA	NA	HTN, DM, HLP, Smoke, CVD	CP, Dyspnea/ NA	NA	NA	8
Mommersteeg P.M.C., et al. (2017) [74]	Netherlands	European	Cross-Sectional	1870	523	NA	1347	NA	Obesity, Smoke, DM, HTN, Lung Disease, DLP, PAD, TIA, Stroke	NA/ CRP, Hb	Statin, ACE-I, Beta-Blocker	NA	9
Rossini R., et al. (2013) [75]	Japan	Western Pacific	Cohort	1206	318	888	NA	780 ± 480	Smoke, HTN, DLP, CAD, DM, MI, Stroke, PCI, CABG, AF, Kidney Disease	NA	NA/ Clopidogrel, Beta-Blocker, Statin, ACE-I or ARB, Aspirin	MACE, Death, MI, Stroke, Bleeding	9
Zeljkovic A., et al. (2014) [76]	USA	Americas	Cross-Sectional	140	40	9	NA	NA	HTN	NA/ TC, TG, LDL, HDL, CRP	NA	NA	9
Reynolds H.R., et al. (2021) [6]	China	Western Pacific	Retro-Cohort	8518	208	865	NA	780	HTN, DM, Smoke, MI, HF, AF, COPD, Depression	NA	NA	NA	9
Qi L.,et al. (2020) [77]	New York	Americas	Cross-Sectional	254	72	NA	48	NA	Smoke, HTN, DM	NA/ TC, TG, HDL, LDL	NA	NA	8
Planer D., et al. (2014) [78]	Sweden	European	Pro-Cohort	6921	197	2245	NA	365	DM, HTN, HLP, Smoke, MI, PCI	NA/ Platelets, Hb, CRP	ASA, Statin, Beta-Blocker, ACE-I, ARB/ Beta-Blocker, Statin, ACE-I or ARB, Aspirin	Death, CV-Death, MI, Bleeding, Revascularization	9
Eggers K.M., et al. (2019) [79]	Canada	Americas	Retro-Cohort	106521	7266	69267	4069	1387	Smoke, HTN, DM, HLP, HF, PVD, Stroke, COPD, Dementia, AF	NA/ GFR, EF	Aspirin, P2Y12-I, Beta-Blocker, RAAS-I, Statin/ Beta-Blocker, Statin, Aspirin, P2Y12-I, RAAS-I	MACE, Death, CV-Death, MI, HF, Stroke	9
Chow B.J., et al. (2015) [80]	Korea	south-east Asia	Pro-Cohort	10418	4706	NA	5712	816 ± 513.33	HTN, DM, DLP, CAD, Smoke	CP, Dyspnea/ NA	Statin, Aspirin/ Statin, Aspirin	NA	9
Choo E.H., et al. (2019) [81]	USA	Americas	Retro-Cohort	13104	396	10871	NA	730	HF, HTN, DM, DLP, CAD, CVA, Smoke, AF	CP, Dyspnea, SBP, DBP/ Cr, CK-MB, Troponin I, Troponin T, HDL, LDL, TG, TC	NA/ Beta-Blocker, Statin, Aspirin, CCB, P2Y12-I, RAAS-I	Death, CV-Death, MI, HF, Cardiogenic Shock, Revascularization	9
Chokshi N.P., et al. (2010) [82]	Sweden	European	Cross-Sectional	1088	106	412	NA	NA	NA	NA	NA	NA	8
Brolin E.B., et al. (2015) [83]	Sweden	European	Cross-Sectional	152	57	NA	58	NA	Smoke, DM, HTN, HLP, CAD	NA	NA	NA	8
Brolin E.B., et al. (2018) [84]	Canada	Americas	Retro-Cohort	115	49	66	NA	90	Smoke, DM, HTN, HLP, CAD	SBP/ GFR, CRP, HDL, LDL, TG, TC	NA	NA	9
Braga J.R., et al. (2019) [85]	Canada	Americas	Retro-Cohort	12814	2254	7904	2656	365	AF, Cancer, Kidney Disease, COPD, Smoke, Dementia, Depression, DM, HLP, HTN, MI, HF, PVD, Stroke	NA/ Cr, EF	NA	Death, CV-Death, MI, HF, Stroke	9
Edlinger M., et al. (2018) [86]	Italy	European	Cross-Sectional	8296	1606	1901	1381	NA	DM, HTN, DLP, Smoke	CP/ HDL, LDL, CRP	NA	NA	8
Dwyer J.P., et al. (2008) [87]	Australia	Western Pacific	Pro-Cohort	824	29	151	NA	360	MI, PCI, CABG, DM, HTN	SBP/ TC, LDL, HDL, TG, Smoke	Aspirin, Clopidogrel, Warfarin, Statin, ACE-I, Beta-Blocker, Nitrate, Diuretic	Death, CV-Death, MI	9
Durmaz E., et al. (2020) [7]	Turkey	European	Pro-Cohort	186	126	60	NA	2920	HTN, DM, HLP, Smoke	NA/ EF, LDL, CRP	Aspirin, ACE-I, ARB, Beta-Blocker, Statin, Nitrates	NA	9
Dreyer R.P., et al. (2020) [88]	USA	Americas	Cohort	677106	16849	269931	NA	365	HF, MI, CVD, Stroke, DM, Kidney Disease, COPD	NA	NA	MACE, Death, MI, HF, Stroke	9
De Ferrari G.M., et al. (2011) [89]	Italy	European	Retro-Cohort	3306	125	125	NA	1095	NA	NA/ LDL, HDL, TG, Cr, CRP, CK-MB	NA/ Beta-Blocker, Statin, ACE-I or ARB, Aspirin, Nitrates, CCB, P2Y12-I, RAAS-I	Death, CV-Death, MI	9
Dasari T.W., et al. (2013) [10]	Spain	European	Retro-Cohort	354	119	103	NA	360 ± 60	HTN, DM, CKD, HF, DLP	NA/ TC, TG, HDL, LDL, Cr, Hb, SBP, DBP	Aspirin, Clopidogrel, Beta-Blocker, Statin, CCB, ACE-I, ARB	NA	9
Lopez Pais J., et al. (2022) [90]	Turkey	European	Retro-Cohort	553	109	412	NA	519 ± 279	HTN, DM, DLP, Smoke, Cancer, AF	SBP/ Troponin T, Hb, Cr	NA	MACE, Death, CV-Death, MI, Stroke, Cardiogenic Shock, Pulmonary Edema, Bleeding	9
Asil S., et al. (2022) [14]	Germany	European	Retro-Cohort	155	77	NA	78	365	DM, HTN, CAD	NA/ CRP, Cr, EF	NA	NA	9
Kruska M., et al. (2021) [91]	Canada	Americas	Retro-Cohort	8322	72	51	NA	NA	CAD, AF, PAD, TIA, HTN, DM, Smoke, DLP	NA/ EF, Troponin I, Cr, GFR, TC, TG, HDL, LDL, Hb	NA	NA	9
Usui E., et al. (2022) [92]	Canada	Americas	Retro-Cohort	301	58	52	NA	365	HTN, DM, DLP, Smoke, Kidney Disease, MI	NA/ TC, LDL, HDL, TG, Troponin	Aspirin, Statin	NA	9
Taslidere B., et al. (2022) [93]	China	Western Pacific	Cross-Sectional	217	20	NA	123	NA	NA	NA/ Troponin, CK-MB	NA	NA	9
Abdu F.A., et al. (2019) [94]	Canada	Americas	Cohort	2029	128	1901	NA	365	HTN, DM, HLP, Smoke, Stroke, AF, HF	SBP, DBP/ EF, CRP, TC, TG, HDH, LDL, CK-MB	NA/ Clopidogrel, Beta-Blocker, Statin, ACE-I or ARB, Aspirin, CCB	MACE, CV-Death, MI, Stroke, HF	9
Adatia F., et al. (2017) [95]	Canada	Americas	Cohort	15058	125	3634	82	90	DM, Kidney Disease, PVD, CVA, HF, COPD, Liver Disease, Cancer, HTN	NA	NA/ Clopidogrel, Beta-Blocker, Statin, ACE-I or ARB, CCB	NA	8
Beigel R., et al. (2013) [96]	New Zealand	Western Pacific	Cohort	959	312	NA	545	810 ± 330	DM, HTN, HLP, CAD	NA/ TC, HDL, LDL, TG, Cr, CRP	NA	MACE, Death, CV-Death,	9
Bainey K.R., et al. (2018) [97]	USA	Americas	Cohort	36214	2092	33836	NA	1825	MI, HF, PVD, Stroke, TIA, Dementia, DM, Kidney Disease, Cancer, AF	NA	NA	Death, CV-Death, MI, Stroke, HF	9
Andreini D., et al. (2017) [98]	Germany	European	Cohort	5010	952	NA	1450	1794 ± 417	HTN, DM, Smoke, DLP	CP/ NA	Statin, Aspirin/ Statin, Aspirin	NA	9
Andersson H.B., et al. (2018) [99]	New Zealand	Western Pacific	Pro-Cohort	4896	298	4239	256	949	HTN, HLP, Smoke, DM, MI, HF, Stroke	NA	NA	Death, CV-Death, Stroke, Cardiogenic Shock	9
Aldous S., et al. (2015) [100]	USA	Americas	Retro-Cohort	10962	171	NA	180	730	MI, HTN, DLP, DM, Smoke, CVA, TIA, PVD	CP/ Troponin	NA/ Clopidogrel, Beta-Blocker, Statin, ACE-I or ARB, Aspirin	Death, CV-Death, MI, HF, Revascularization	8
Soofi M., et al. (2020) [101]	USA	Americas	Retro-Cohort	1535	229	1110	196	6935	HTN, HLP, DM, CKD, Smoke, PVD	NA	NA	Death	9
Sharaf B., et al. (2013) [102]	Sweden	European	Pro-Cohort	917	228	350	339	3394.5	HTN, DM, Smoke, HLP, CAD	CP/ SBP, DBP	Aspirin, ACE-I, Beta-Blocker, Statin	NA	8
Hanson C.A., et al. (2020) [103]	USA	Americas	Cohort	1579	378	472	NA	2190	DM, HTN, HLP, HF, PVD, CKD	CP/ NA	NA	NA	8
Gulati M., et al. (2009) [104]	Italy	European	Pro-Cohort	7603	222	NA	318	1898	CAD, HTN, DM, Smoke	SBP, DBP/ HDL, LDL	ASA	Death, MI, Stroke, HF	9
Galway S., et al. (2017) [105]	Canada	Americas	Cohort	15058	1088	4893	1554	90	DM, Kidney Disease, PVD, CVA, HF, COPD, Cancer, Liver Disease	NA	ACE-I, ARB, Beta-Blocker, Statin, CCB, Clopidogrel/ Clopidogrel, Beta-Blocker, Statin, Nitrates	NA	8
Feuchtner G.M., et al. (2010) [8]	Sweden	European	Cross-Sectional	1060	353	334	NA	NA	DM, HTN, HLP, Smoke	NA	NA	NA	9
Abdelmonem Y.Y., et al. (2017) [106]	Egypt	Eastern Mediterranean	Case-Control	200	9	9	NA	NA	Smoke, DM, HTN, DLP, CAD, PCI, HF, PVD, Kidney Disease, Stroke	NA/ Troponin I, CK-MB, TC, LDL, HDL, TG, Hb, Cr	NA/ Beta-Blocker, ACE-I or ARB, CCB,	NA	9
Paolisso P., et al. (2021) [107]	Italy	European	Cross-Sectional	2795	239	2450	NA	NA	Smoke, HTN, DLP, DM, MI, Stroke, COPD, PAD, AF	SBP, DBP/ Hb, Cr, TC, LDL, TG	NA	NA	9
Barr P.R., et al. (2017) [108]	New Zealand	Western Pacific	Cohort	2419	302	1768	NA	792 ± 485	Smoke, DM, CVD, HF	NA/ LDL, Troponin	NA/ Clopidogrel, Beta-Blocker, Statin, ACE-I or ARB, Aspirin	NA	9
Ballesteros D., et al. (2019) [109]	Spain	European	Retro-Cohort	12899	622	9241	NA	NA	Smoke, HTN, CAD, DLP, DM, MI, Stroke, PAD, HF, COPD, Kidney Disease	SBP, DBP/ Cr, TC, HDL, TG, Hb	Aspirin, Clopidogrel, Nitrates, Beta-Blocker, CCB, ACE-I, ARB	NA	9
Kuneman J.H., et al. (2022) [110]	Netherlands	European	Cross-Sectional	5083	856	2191	NA	NA	HTN, HLP, Smoke	CP, Dyspnea/ NA	NA	NA	9
Stepien K., et al. (2022) [111]	Poland	European	Retro-Cohort	1011	72	939	NA	135	DM, HTN, DLP, Smoke, MI, Stroke	NA/ Hb, Cr, GFR, Troponin, CK-MB	NA/ Beta-Blocker, Statin, ACE-I or ARB, Aspirin, P2Y12-I	NA	9
Diaz-Arocutipa C., et al. (2021) [112]	Peru	Americas	Pro-Cohort	161	28	133	NA	NA	HTN, DM, CAD, DLP, CKD, PCI	CP, Dyspnea/ EF	NA	NA	9
Zhang H.W., et al. (2021) [9]	China	Western Pacific	Pro-Cohort	10940	1290	7801	NA	1200	HTN, DLP, DM, Smoke, CAD	NA/ TC, TG, LDL, HDL, Cr, Hb	Aspirin, Beta-Blocker, CCB, ACE-I, ARB	NA	9
Soman P., et al. (2006) [113]	Mass	Americas	Cross-Sectional	91	36	NA	55	NA	HTN, HLP, DM, CAD, Smoke	NA	NA	NA	8
Radico F., et al. (2015) [114]	Italy	European	Pro-Cohort	2861	956	NA	1905	2190 ± 162	HTN, DM, DLP, Smoke, CAD	NA	Statin, ACE-I, ARB/ Beta-Blocker, Statin, ACE-I or ARB, Anti-Platelet	NA	8
Maddox T.M., et al. (2010) [115]	USA	Americas	Retro-Cohort	37674	8384	20899	8391	365	HTN, HLP, DM, Smoke, Obesity, HF, COPD, CVD, PAD, Depression, CKD	CP/ NA	Statin, ACE-I, ARB, Beta-Blocker/ Beta-Blocker, Statin, ACE-I or ARB	NA	9
Khajouei A.S., et al. (2019) [116]	Iran	Eastern Mediterranean	Retro-Cohort	527	103	NA	362	NA	HTN, HLP, DM, Smoke	NA	NA	NA	8
Ramanath V.S., et al. (2009) [117]	Michigan	Americas	Cohort	3514	123	2141	NA	180	HTN, HLP, DM, Smoke, MI, CAD, PVD, Stroke	NA	NA/ Clopidogrel, Beta-Blocker, Statin, ACE-I or ARB, Aspirin	NA	9
Johnson B.D., et al. (2006) [118]	USA	Americas	Cohort	936	412	261	NA	365	HTN, DLP, DM, Smoke, CAD, Obesity, Depression	NA	NA	NA	9
Vranken N.P.A., et al. (2020) [119]	Netherlands	European	Retro-Cohort	7693	402	7291	NA	1642.5 ± 1162.59	HTN, HLP, DM, Smoke, PAD, Kidney Disease, CVA, MI, PCI, CABG	NA/ CK-MB, CRP, Troponin, Cr	NA	NA	9
Janosi A. et al. (2018) [120]	Hungary	European	Cohort	45223	2003	43220	NA	730	NA	NA	NA/ Beta-Blocker, Statin, Aspirin, RAAS-I,	NA	9
Fernandez M.C., et al. (2016) [121]	Spain	European	Retro-Cohort	4499	643	3856	NA	450 ± 298	NA	NA	NA/ Statin	NA	9
Duran B.A., et al. (2018) [122]	Spain	European	Cohort	521	22	87	NA	30	NA	NA	NA	NA	9
Donati F., et al. (2019) [123]	Italy	European	Cohort	1990	186	1804	NA	588 ± 387	NA	NA	NA	NA	9
Coronel B.I., et al. (2018) [124]	Spain	European	Cohort	294	98	196	NA	420	NA	NA	NA	NA	9
Bossard M., et al. (2019) [125]	Canada	Americas	Cohort	25086	1599	21888	NA	30	NA	NA	NA	NA	9
Li S., et al. (2022) [126]	China	Western Pacific	Cross-Sectional	631	289	132	101	NA	HTN, HLP, DM	NA/ Hb, GFR, TC, TG, HDL, LDL, EF	Beta-Blocker	NA	9
Vorobeva D.A., et al. (2022) [127]	Russian	European	Case–Control	1120	16	21	NA	NA	HTN, DLP, DM, CAD, Smoke, Stroke, PAD	NA/ Troponin-I, GFR	NA	NA	9
Zhao X., et al. (2022) [128]	China	Western Pacific	Cross-Sectional	105	21	30	NA	NA	HTN, DLP, DM, Smoke, Obesity	NA/ EF	NA	NA	8
Kilic S., et al. (2020) [129]	Turkey	European	Pro-Cohort	1793	109	1517	NA	NA	HTN, HLP, DM, Smoke, CAD	SBP, DBP/ NA	NA	NA	9
Hjort M., et al. (2023) [130]	Sweden	European	Retro-Cohort	18624	107	2755	NA	NA	HTN, DLP, DM, Smoke, MI, PCI, CABG, HF, Stroke, PAD, Kidney Disease	NA/ GFR, Troponin-T, CRP	Aspirin, Beta-Blocker, RAAS-I, Statin	NA	9

Summarizes key details of included studies: first author, country, design, sample size, follow-up, and quality score based on the Newcastle-Ottawa Scale

*Abbreviations:* *NA* Not Available, *Pro-Cohort* Prospective Cohort, *Retro-Cohort* Retrospective Cohort, *BMI* Body Mass Index, *DM* Diabetes Mellitus, *HTN* Hypertension, *DLP* Dyslipidemia, *CAD* Coronary Artery Disease, *MI* Myocardial Infarction, *PCI* Percutaneous Coronary Intervention, *CABG* Coronary Artery Bypass Graft, *AF* Atrial Fibrillation, *HF* Heart Failure, *PVD* Peripheral Vascular Disease, *CVA* Cerebro-Vascular Accident, *TIA* Transient Ischemic Attack, *COPD* Chronic Obstructive Pulmonary Disease, *CKD* Chronic Kidney Disease

**Table 3:** Comparison of demographics and clinical characteristics in MINOCA, MIOCA and control

**Variable**		Studies (n)	Pooled Prevalence of each Variable in Study Groups				**MIOCA **Vs **MINOCA **(SMD^a^/OR (95% CI))	*P*-value for SMD=0/ OR=1	Cochrane Q	I^2^ (%)	*P*-value for Egger Test
			Sample Size/ Total **MIOCA**	Pooled Prevalenc in **MIOCA **(%)	Sample Size/ Total** MINOCA**	Pooled Prevalenc in **MINOCA **(%)					
**Demographics**	**Age**^a^	88		63.72±5.62		61.52±5.17	0.22 (0.15, 0.29)	0.001>	25031.53	99.7	0.432
	**BMI**^a^	45		27.17±2.08		27.07±2.46	0.03 (-0.03, 0.09)	0.376	1369.57	96.8	0.001>
	**Male**^**b**^	76	1485903/ 2259128	65.89 (65.83, 65.96)	335199/ 701828	47.84 (47.72, 47.95)	2.36 (2.12, 2.62)	0.001>	9882.49	99.2	0.055
**Medical History**^**b**^	**DM**	81	675370/ 2166294	30.65 (30.58, 30.71)	87334/ 322703	26.49 (26.34, 26.65)	1.62 (1.49, 1.76)	0.001>	1811.84	95.6	0.001>
	**HTN**	79	1325207/ 1856085	71.76 (71.7, 71.83)	219285/ 303443	72.82 (72.66, 72.98)	1.26 (1.12, 1.42)	0.001>	4270.99	98.2	0.61
	**Smoker**	73	228295/ 572794	38.63 (38.51, 38.76)	20513/ 63095	31.53 (31.17, 31.89)	1.51 (1.30, 1.75)	0.001>	3084.77	97.7	0.108
	**DLP**	16	1098395/ 1656737	66.52 (66.45, 66.6)	187607/ 290986	64.7 (64.52, 64.88)	1.54 (1.38, 1.73)	0.001>	2479.49	97.2	0.71
	**Obesity**	13	561018/ 1440793	38.71 (38.63, 38.79)	113690/ 255843	44.34 (44.15, 44.53)	0.90 (0.80, 1.019)	0.101	124.63	90.4	0.390
	**Angina**	6	4622/ 26274	15 (14.57, 15.43)	331/ 1558	20.15 (18.18, 22.19)	1.2 (0.63, 2.29)	0.582	101.36	95.1	0.551
	**CAD**	11	50777/ 453231	10.8 (10.71, 10.89)	1386/ 26644	4.15 (3.91, 4.41)	2.89 (1.89, 4.42)	0.001>	260.46	96.2	0.274
	**MI**	29	479766/ 1961092	24.09 (24.03, 24.15)	47619/ 281726	16.22 (16.08, 16.35)	2.40 (2.06, 2.80)	0.001>	644.48	95.7	0.259
	**Stroke**	27	23466/ 803615	2.54 (2.5, 2.58)	1703/ 54452	2.52 (2.39, 2.67)	1.02 (0.80, 1.29)	0.874	405.61	93.6	0.437
	**PCI**	17	441281/ 1568528	26.11 (26.04, 26.18)	71111/ 253237	27.22 (27.04, 27.39)	2.94 (2.16, 3.99)	0.001>	443.27	96.4	0.085
	**CABG**	14	304885/ 1565963	18.15 (18.09, 18.21)	3451/ 252916	0.92 (0.87, 0.96)	7.31 (2.68, 19.92)	0.001>	873.89	98.5	0.742
	**AF**	16	5535/ 84192	5.22 (5.07, 5.38)	1320/ 11059	11.07 (10.48, 11.67)	0.59 (0.41, 0.85)	0.005	264.98	94.3	0.873
	**HF**	22	189815/ 1748594	10.45 (10.4, 10.49)	29696/ 286557	10.06 (9.95, 10.18)	0.80 (0.69, 0.93)	0.004	439.60	95.2	0.056
	**PVD**	24	176732/ 1459835	11.58 (11.53, 11.63)	23664/ 266861	8.52 (8.41, 8.63)	1.51 (1.28, 1.77)	0.001>	203.18	88.7	0.38
	**CVA**	10	159617/ 1303696	12.18 (12.12, 12.23)	24537/250226	9.64 (9.52, 9.75)	1.32 (1.19, 1.46)	0.001>	18.70	51.9	0.101
	**TIA**	4	1782/ 6182	21.35 (20.33, 22.4)	170/ 583	22.88 (19.52, 26.41)	1.15 (0.89, 1.5)	0.283	2.37	0	0.235
	**COPD**	14	73151/ 639736	9.52 (9.45, 9.6)	8066/ 54017	13.21 (12.93, 13.5)	0.67 (0.55, 0.83)	0.001>	473.65	97.3	0.790
	**Chronic Lung Disease**	5	213420/ 1259418	16.9 (16.84, 16.97)	47047/ 237814	19.43 (19.27, 19.59)	0.74 (0.42, 1.32)	0.309	31.11	87.1	0.727
	**CKD**	11	7561/ 93565	7.65 (7.48, 7.82)	1533/ 16299	8.75 (8.31, 9.2)	1.06 (0.88, 1.27)	0.539	37.55	73.4	0.948
	**Kidney Disease**	14	45753/ 626206	6.84 (6.77, 6.9)	2536/ 37111	6.03 (5.78, 6.28)	1.06 (0.80, 1.39)	0.686	201.53	93.5	0.639
	**Liver Disease**	2	442/ 14242	2.6 (2.34, 2.87)	63/ 1133	5.02 (3.8, 6.39)	0.64 (0.49, 0.84)	0.001	0.13	0	0.001>
	**Depression**	6	8708/ 36588	22.06 (21.63, 22.48)	3805/ 11427	31.34 (30.49, 32.2)	0.82 (0.62, 1.09)	0.184	40.09	87.5	0.165
	**Dementia**	4	312/ 114982	0.19 (0.16, 0.21)	53/ 13324	0.29 (0.21, 0.4)	0.86 (0.45, 1.67)	0.667	8.17	63.3	0.658
	**Cancer**	10	3750/ 168193	1.85 (1.79, 1.92)	571/ 17509	2.73 (2.49, 2.99)	0.72 (0.51, 1.002)	0.051	92.30	90.2	0.885
**Family History**^**b**^	**CAD**	24	13504/ 55168	22.63 (22.28, 22.98)	4314/ 14793	28.04 (27.31, 28.78)	1.21 (1.07, 1.37)	0.003	69.76	67	0.21

**Variable**		Studies (n)	Pooled Prevalence of each Variable in Study Groups				**MINOCA **Vs **Control **(SMD^a^/OR (95% CI))	*P*-value for SMD=0/ OR=1	Cochrane Q	I^2^ (%)	*P*-value for Egger Test
			Sample Size/ Total **MINOCA**	Pooled Prevalenc in **MINOCA**	Sample Size/ Total **Control**	Pooled Prevalenc in **Control**					
**Demographics**	**Age**^**a**^	40		59.87±4.94		55.05±7.01	0.40 (0.31, 0.49)	0.001>	1415.88	97.2	0.06
	**BMI**^**a**^	24		27.09±1.9		26.48±2.1	0.1 (0.04, 0.15)	0.001	147.57	84.4	0.455
	**Male**^**b**^	36	27734/ 47333	60.61 (60.17, 61.06)	28672/ 57100	50.94 (50.53, 51.36)	1.25 (1.08, 1.45)	0.003	651.56	94.6	0.383
**Medical History**^**b**^	**DM**	38	9162/ 49062	17.03 (16.7, 17.37)	7462/ 59852	10.79 (10.54, 11.05)	1.52 (1.35, 1.72)	0.001>	202.08	81.2	0.45
	**HTN**	39	28711/ 49003	59.45 (59.01, 59.89)	26470/ 59787	43.9 (43.5, 44.31)	1.89 (1.60, 2.24)	0.001>	954.16	96	0.078
	**Smoker**	33	15437/ 47425	31.44 (31.02, 31.87)	16926/ 57354	28.4 (28.03, 28.77)	1.21 (1.10, 1.32)	0.001>	169.45	81.1	0.127
	**DLP**	30	17037/ 2620	55.08 (54.54, 55.62)	17538/ 33118	52.53 (51.99, 53.08)	1.97 (1.57, 2.46)	0.001>	681.05	95.7	0.091
	**Obesity**	3	4332/ 11039	39.17 (38.26, 40.08)	4247/ 11445	36.65 (35.77, 37.53)	1.29 (0.91, 1.81)	0.149	42.15	95.3	0.193
	**Angina**	1	114/ 228	50 (43.33, 56.67)	186/ 339	54.87 (49.4, 60.25)	0.82 (0.59, 1.15)	0.255	NA	NA	NA
	**CAD**	1	6/ 77	7.79 (2.91, 16.19)	7/ 78	NA	0.86 (0.27, 2.68)	0.791	NA	MA	NA
	**MI**	6	506/ 4023	11.71 (10.76, 12.69)	388/ 5549	7.68 (6.99, 8.4)	2.71 (1.71, 4.31)	0.001>	21.01	76.2	0.036
	**Stroke**	5	563/ 9863	5.02 (4.57, 5.48)	281/ 7231	NA	1.76 (1.29, 2.40)	0.001>	7.81	48.8	0.704
	**PCI**	1	4/ 23	17.39 (4.95, 38.78)	75/ 184	40.76 (33.59, 48.23)	0.31 (0.1, 0.94)	0.038	NA	NA	NA
	**CABG**	2	2/ 251	0.23 (0, 1.72)	12/ 523	1.01 (0.27, 2.13)	1.42 (0.06, 34.28)	0.829	2.33	57	0.001>
	**AF**	2	501/ 2276	21.39 (19.68, 23.16)	553/ 2722	20.04 (18.54, 21.58)	1.09 (0.95, 1.25)	0.199	0.15	0	0.001>
	**HF**	6	1994/ 21342	NA	1833/ 19158	7.56 (7.19, 7.94)	1.41 (1.03, 1.94)	0.033	62.51	92	0.317
	**PVD**	7	1491/ 21569	5.78 (5.47, 6.1)	837/ 19360	3.67 (3.4, 3.94)	1.93 (1.76, 2.11)	0.001>	0.97	0	0.412
	**CVA**	4	1133/ 11820	9.17 (8.65, 9.7)	780/ 12439	5.9 (5.48, 6.33)	1.58 (1.44, 1.74)	0.001>	1.16	0	0.002
	**TIA**	NA	NA	NA	NA	NA	NA	NA	NA	NA	NA
	**COPD**	4	3049/ 19037	15.2 (14.69 15.72)	2174/ 17277	11.54 (11.06, 12.02)	1.63 (1.2, 2.21)	0.002	45.49	93.4	0.462
	**Chronic Lung Disease**	2	77/ 546	13.11 (10.28, 16.2)	213/ 1531	13.84 (12.14, 15.62)	0.71 (0.16, 3.13)	0.65	1.66	39.8	0.001>
	**CKD**	3	1252/ 10867	11.45 (10.86, 12.06)	988/ 11243	8.7 (8.19, 9.23)	1.36 (1.24, 1.48)	0.001>	0.25	0	0.721
	**Kidney Disease**	1	23/ 1133	1.77 (1.04, 2.66)	23/ 2161	0.8 (0.42, 1.28)	2.09 (1.17, 3.74)	0.013	NA	NA	NA
	**Liver Disease**	1	63/ 1133	5.02 (3.8, 6.39)	106/ 2161	4.55 (3.68, 5.5)	1.24 (0.9, 1.71)	0.188	NA	NA	NA
	**Depression**	2	3629/ 10638	32.13 (31.25, 33.02)	4089/ 11047	34.59 (33.7, 35.48)	0.87 (0.71, 1.06)	0.161	2.51	60.2	0.001>
	**Dementia**	2	49/ 9520	0.4 (0.28, 0.54)	18/ 6725	0.17 (0.08, 0.29)	3.1 (1.1, 8.77)	0.033	1.46	31.6	0.001>
	**Cancer**	4	398/ 10653	3.24 (2.9, 3.59)	263/ 8886	2.39 (2.07, 2.73)	1.81 (1.39, 2.36)	0.001>	5.48	45.2	0.073
**Family History**^**b**^	**CAD**	14	8804/ 25799	33.7 (33.12, 34.29)	11731/ 34485	33.17 (32.67, 33.67)	1.15 (0.97, 1.38)	0.114	156.68	91.7	0.10

Compares demographic and clinical features (e.g., age, BMI, smoking, hypertension) in MINOCA, MIOCA, and control groups, with statistical measures (SMD, OR)

*Abbreviations:* *NA* Not Available, *BMI* Body Mass Index, *DM* Diabetes Mellitus, *HTN* Hypertension, *DLP* Dyslipidemia, *CAD* Coronary Artery Disease, *MI* Myocardial Infarction, *PCI* Percutaneous Coronary Intervention, *CABG* Coronary Artery Bypass Graft, *AF* Atrial Fibrillation, *HF* Heart Failure, *PVD* Peripheral Vascular Disease, *CVA* Cerebro-Vascular Accident, *TIA* Transient Ischemic Attack, *COPD* Chronic Obstructive Pulmonary Disease, *CKD* Chronic Kidney Disease

An SMD of zero means no considerable effects. SMDs greater than zero indicate direct effect-and SMDs less than zero indicate inverse effect [19]. Cohen offered the following guidelines for interpreting the magnitude of the SMD in the social sciences: small-SMD = 0.2; medium-SMD = 0.5; and large-SMD = 0.8 [126]

^a^polled effect for quantitative values-was reported by SMD

^b^Polled effect for qualitative values-was reported by OR

**Table 4:** Comparison of clinical presentation and laboratory findings in MINOCA, MIOCA and control

**Variable**		Studies (n)	Pooled Prevalence of each Variable in Study Groups				**MIOCA **Vs **MINOCA** (SMD^a,b^/OR (95% CI))	*P*-value for SMD=0/ OR=1	Cochrane Q	I^2^ (%)	*P*-value for Egger Test
			Sample Size/ Total **MIOCA**	Pooled Prevalenc in **MIOCA**(%)	Sample Size/ Total** MINOCA**	Pooled Prevalenc in **MINOCA**(%)					
**Symptoms**^**c**^	**SA**	6	341005/ 153104	21.01 (20.95, 21.08)	176697/ 632172	27.28 (27.17, 27.39)	0.65 (0.42, 1.02)	0.059	8788.96	99.9	0.837
	**Syncope**	1	108/ 151	71.52 (63.62, 78.56)	14/ 29	48.28 (29.45, 67.47)	2.69 (1.2, 6.05)	0.017	NA	NA	NA
	**Chest Pain**	10	15658/ 24474	64.15 (63.54, 64.75)	7728/ 11576	67.05 (66.18, 67.92)	1.09 (0.76, 1.57)	0.634	105.63	91.5	0.221
	**CP Typical**	4	797/ 3170	24.79 (23.3, 26.31)	562/ 3632	14.83 (13.68, 16)	1.66 (1.25, 2.21)	0.001>	11.21	73.2	0.125
	**CP Atypical**	5	166168/ 1530547	10.57 (10.52, 10.62)	195597/ 626390	31.06 (30.95, 31.18)	0.58 (0.47, 0.72)	0.001>	1551.0	99.7	0.399
	**No CP**	NA	NA	NA	NA	NA	NA	NA	NA	NA	NA
	**Non-Cardiac CP**	3	562/ 3107	14.34 (13.13, 15.6)	567/ 3366	14.81 (13.62, 16.03)	1.1 (0.59, 2.04)	0.769	20.05	90	0.852
	**Non-Anginal CP**	2	88321/ 275334	32.04 (31.87, 32.22)	116313/ 386235	30.07 (29.93, 30.22)	0.96 (0.66, 1.39)	0.817	3.11	67.9	0.001>
	**Dyspnea**	6	795/ 4346	16.78 (15.68, 17.91)	538/ 3265	15.85 (14.58, 17.16)	0.64 (0.37, 1.11)	0.115	33.84	85.2	0.45
**ECG data**^**c**^	**STE**	32	210205/ 1440978	13.43 (13.37, 13.48)	5334/ 254920	0.92 (0.87, 0.96)	3.39 (1.96, 5.87)	0.001>	7174.19	99.6	0.028
	**STD**	8	6602/ 34101	18.66 (18.25, 19.08)	637/ 2780	21 (19.48, 22.56)	1.19 (0.74, 1.93)	0.477	89.23	92.2	0.597
	**EF (%)**	23	NA	NA	NA	NA	-0.38 (-0.5, -0.27)	0.001>	1701.19	98.7	0.224
**Vital Sign**^**a,b**^	**SBP **	14	NA	NA	NA	NA	0.01 (-0.06, 0.07)	0.815	45.40	71.4	0.667
	**DBP**	9	NA	NA	NA	NA	-0.01 (-0.07, 0.06)	0.892	15.20	47.4	0.961
**Laboratory data**^**a,b**^	**LDL (mg/dl)**	26	NA	NA	NA	NA	0.18 (0.36, 0.002)	0.053	968.35	97.4	0.079
	**HDL (mg/dl)**	19	NA	NA	NA	NA	-0.25 (-0.38, -0.12)	0.001>	280.66	93.6	0.972
	**TG (mg/dl)**	19	NA	NA	NA	NA	0.02 (-0.09, 0.14)	0.702	159.84	88.7	0.919
	**TC (mg/dl)**	17	NA	NA	NA	NA	0.17 (0.37, 0.02)	0.043	423.92	96.2	0.353
	**GFR**	18	NA	NA	NA	NA	-0.08 (-0.13, -0.02)	0.84	104.21	83.7	0.078
	**CRP (mg/dl)**	7	NA	NA	NA	NA	-0.01 (-0.08, 0.1)	0.048	9.78	38.7	0.146
	**Cr (mg/dl)**	16	NA	NA	NA	NA	0.11 (0.03, 0.18)	0.062	50.58	70.3	0.298
	**Hb (mg/dl)**	NA	NA	NA	NA	NA	NA	NA	NA	NA	NA
	**Platelets (10**^**9**^**/L)**	3	NA	NA	NA	NA	-0.02 (-0.14, 0.09)	0.667	0.34	0	0.35
	**Troponin (IU/L)**	5	NA	NA	NA	NA	-0.37 (-1.28, 0.53)	0.422	555.2	99.3	0.695
	**Troponin-T (IU/L)**	6	NA	NA	NA	NA	NA	0.042	1816.88	99.7	0.298
	**Troponin-I (IU/L)**	6	NA	NA	NA	NA	0.23 (-0.89, 1.35)	0.687	728.32	99.3	0.978
	**CK-MB (IU/L)**	5	NA	NA	NA	NA	-0.25 (-0.16, 0.35)	0.001>	3.32	0	0.806
	**Fibrinogen (g/L) **	3	NA	NA	NA	NA	-0.42 (-0.26, 0.58)	0.001>	1.14	0	0.006
	**BNP (ng/L)**	1	NA	NA	NA	NA	-0.42 (-0.14, 0.71)	0.003	NA	NA	NA

**Variable**		Studies (n)	Pooled Prevalence of each Variable in Study Groups				**MINOCA **Vs **Control **(SMD^a,b^/OR (95% CI))	*P*-value for SMD=0/ OR=1	Cochrane Q	I^2^ (%)	*P*-value for Egger Test
			Sample Size/ Total **MINOCA**	Pooled Prevalenc in **MINOCA**	Sample Size/ Total **Control**	Pooled Prevalenc in **Control**					
**Symptoms**^**c**^	**SA**	1	297/ 8384	3.54 (3.16, 3.96)	281/ 8391	3.35 (2.97, 3.76)	1.06 (0.9, 1.25)	0.492	NA	NA	NA
	**Syncope**	NA	NA	NA	NA	NA	NA	NA	NA	NA	NA
	**Chest Pain**	6	9808/ 15644	62.98 (62.22, 63.74)	11052/ 16513	67.1 (66.38, 67.82)	0.88 (0.7, 1.09)	0.239	56.62	91.2	0.505
	**CP Typical**	5	1993/ 23371	8.45 (8.09, 8.81)	2508/ 30387	8.18 (7.87, 8.49)	0.87 (0.7, 1.1)	0.244	37.11	89.2	0.185
	**CP Atypical**	4	8112/ 18665	43.44 (42.73, 44.15)	10784/ 24675	43.76 (43.14, 44.38)	0.81 (0.67, 0.99)	0.038	29.73	89.9	0.178
	**No CP**	2	974/ 2650	36.72 (34.89, 38.57)	1268/ 3299	38.43 (36.77, 40.09)	0.88 (0.56, 1.4)	0.6	17.68	94.3	0.001>
	**Non-Cardiac CP**	3	4050/ 16967	23.4 (22.77, 24.04)	6666/ 22826	28.92 (28.33, 29.51)	0.66 (0.43, 1.03)	0.068	41.18	95.1	0.787
	**Non-Anginal CP**	1	202/ 1698	11.9 (10.39, 13.53)	183/ 1849	9.9 (8.57, 11.35)	1.23 (0.99, 1.52)	0.056	NA	NA	NA
	**Dyspnea**	5	1845/ 22442	7.68 (7.33, 8.04)	1644/ 29121	5.47 (5.21, 5.74)	1.43 (1.29, 1.58)	0.001>	5.81	31.2	0.045
**ECG data**^**c**^	**STE**	1	15/ 171	8.77 (4.99, 14.06)	20/ 180	11.11 (6.92, 16.64)	0.77 (0.38, 1.56)	0.466	NA	NA	NA
	**STD**	1	10/ 171	5.85 (2.84, 10.49)	10/ 180	NA	1.07 (0.43, 2.6)	0.906	NA	NA	NA
	**EF (%)**	5	NA	NA	NA	NA	-0.07 (-0.19, 0.06)	0.281	1.02	0	0.833
**Vital Sign**^**a,b**^	**SBP **	5	NA	NA	NA	NA	0.15 (0.07, 0.23)	0.001>	4.93	18.9	0.15
	**DBP**	3	NA	NA	NA	NA	0.04 (-0.1, 0.19)	0.56	1.24	0	0.985
**Laboratory data**^**a,b**^	**LDL (mg/dl)**	12	NA	NA	NA	NA	-0.02 (-0.13, 0.1)	0.78	54.69	79.9	0.483
	**HDL (mg/dl)**	10	NA	NA	NA	NA	-0.2 (-0.28, -0.12)	0.001>	15.05	40.2	0.608
	**TG (mg/dl)**	7	NA	NA	NA	NA	0.02 (-0.2, 0.24)	0.873	32.95	81.8	0.08
	**TC (mg/dl)**	6	NA	NA	NA	NA	-0.01 (-0.24, 0.22)	0.93	26.79	81.3	0.876
	**GFR**	5	NA	NA	NA	NA	-0.34 (-0.6, -0 1)	0.008	121.64	96.7	0.934
	**CRP (mg/dl)**	6	NA	NA	NA	NA	0.28 (0.08, 0.48)	0.005	17.79	71.9	0.163
	**Cr (mg/dl)**	4	NA	NA	NA	NA	0.06 (-0.1, 0.21)	0.462	5.35	44	0.219
	**Hb (mg/dl)**	4	NA	NA	NA	NA	-0.04 (-0.21, 0.12)	0.619	1.06	0	0.984
	**Platelets (10**^**9**^**/L)**	2	NA	NA	NA	NA	0.01 (-0.19, 0.19)	0.982	0.83	0	0.001>
	**Troponin (IU/L)**	NA	NA	NA	NA	NA	NA	NA	NA	NA	NA
	**Troponin-T (IU/L)**	1	NA	NA	NA	NA	-0.05 (-0.22, 0.11)	0.537	NA	NA	NA
	**Troponin-I (IU/L)**	1	NA	NA	NA	NA	2.14 (1.6, 2.67)	0.001>	NA	NA	NA
	**CK-MB (IU/L)**	1	NA	NA	NA	NA	-0.21 (-0.58, 0.16)	0.263	NA	NA	NA
	**Fibrinogen (g/L) **	1	NA	NA	NA	NA	0.69 (0.31, 1.07)	0.001>	NA	NA	NA
	**BNP (ng/L)**	1	NA	NA	NA	NA	0.57 (0.29, 0.86)	0.001>	NA	NA	NA

Compares differences in clinical presentations (e.g., chest pain, dyspnea) and lab findings (e.g., LDL, CRP) across the three groups, supported by statistical metrics

*Abbreviations:* *NA* Not Available, *SA* Stable Angina, *CP* Chest Pain, *STE* ST Elevation, *STD* ST Depression, *SBP* Systolic Blood Pressure, *DBP* Diastolic Blood Pressure, *LDL* Low-Density Lipoprotein, *HDL* High-Density Lipoprotein, *TG* Triglyceride, *TC* Total Cholesterol, *GFR* Glomerular Filtration Rate, *CRP* C-Reactive Protein, *Cr* Creatinine, *Hb* Hemoglobin, *IU/L* International Unit per Liter, *CK-MB* Creatinine Kinase-Myocardial Infarction, *BNP* Brain Natriuretic Peptide, *EF* (ventricular) Ejection Fraction

a = polled effect for quantitative values, was reported by SMD (An SMD of zero means no considerable effects. SMDs greater than zero indicate direct effect, and SMDs less than zero indicate inverse effect [19]. Cohen offered the following guidelines for interpreting the magnitude of the SMD in the social sciences: small, SMD = 0.2; medium, SMD = 0.5; and large, SMD = 0.8 [126]).

b = polled effect for quantitative values-was reported by SMD

c = Polled effect for qualitative values-was reported by OR

*P* values less than 0.05 were considered statistically significant

**Table 5:** Comparison of medication prescription at admission and discharge in MINOCA, MIOCA and Control

**Variable**			Studies (n)	Pooled Prevalence of each Variable in Study Groups				**MIOCA **Vs **MINOCA** (OR (95% CI))	*P*-value for OR=1	Cochrane Q	I^2^ (%)	*P*-value for Egger Test
				Sample Size/ Total **MIOCA**	Pooled Prevalenc in **MIOCA **(%)	Sample Size/ Total** MINOCA**	Pooled Prevalenc in **MINOCA **(%)					
**Treatment at Admission**^a^	**Routine Drugs**	**Statin**	16	737681/ 1379552	53.33 (53.25, 53.42)	121714/ 258800	23.94 (23.08, 24.8)	1.38 (1.17, 1.62)	0.001>	429.79	96.5	0.516
		**β-blockers**	18	883140/ 1389729	63.62 (63.54, 63.7)	137153/ 260314	52.65 (52.46, 52.84)	1.51 (1.22, 1.85)	0.001>	1165.45	98.5	0.786
		**CCB**	8	7003/ 33945	19.44 (19.02, 19.87)	1916/ 9967	18.53 (17.76, 19.31)	1.06 (0.84, 1.35)	0.614	43.22	83.8	0.812
	**Anti-Platelet**	**Aspirin**	16	1131826/ 1432719	80.12 (80.05, 80.18)	187387/ 261664	71.91 (71.73, 72.08)	1.28 (0.85, 1.94)	0.242	3505.2	99.6	0.09
		**Clopidogrel**	9	742540/ 1317415	55.68 (55.59, 55.76)	59932/ 242490	24.43 (24.26, 24.61)	1.67 (0.4, 6.88)	0.478	2920.43	99.7	0.249
		** P2Y12-Inhibitor**	5	3995/ 100355	3.49 (3.38, 3.61)	773/ 16671	4.16 (3.85, 4.47)	1.53 (1.02, 2.31)	0.041	58.66	93.2	0.503
		** Any Anti-Platelet**	2	6348/ 7878	81.05 (80.17, 81.91)	637/ 1018	62.68 (59.67, 65.64)	2.32 (2.01, 2.68)	0.001>	0.05	0	0.001>
	**Anti-Coagulant**	** Warfarin**	1	118/ 3630	3.25 (2.7, 3.88)	15/ 200	7.5 (4.26, 12.07)	0.41 (0.24, 0.72)	0.002	NA	NA	NA
		** Heparin**	1	1556/ 2761	56.36 (54.48, 58.22)	54/ 142	38.03 (30.02, 46.55)	2.1 (1.49, 2,98)	0.001>	NA	NA	NA
		** LMWH**	1	1626/ 2761	58.89 (57.03, 60.73)	94/ 142	66.2 (57.79, 73.91)	0.73 (0.51, 1.04)	0.085	NA	NA	NA
		**Any-Anti-Coagulant**	3	2899/ 95812	3.02 (2.91, 3.12)	498/ 9527	5.2 (4.76, 5.66)	0.56 (0.51, 0.62)	0.001>	1.07	0	0.23
	**ACE-I and/or ARB**	** RAAS-I (ACE-I + ARB)**	3	7781/ 22509	34.24 (33.62, 34.86)	683/ 1985	34.28 (32.3, 36.4)	1.17 (0.7, 1.95)	0.546	25.37	92.1	0.296
		** ACE-I**	6	8150/ 23744	34.01 (33.41, 34.62)	2502/ 9507	25.96 (25.07, 26.85)	1.33 (1.09, 1.63)	0.005	26.83	81.4	0.142
		** ARB**	2	1952/ 7952	24.43 (23.49, 25.39)	265/ 1319	19.36 (17.22, 21.6)	5.82 (0.16, 214.74)	0.339	6.71	85.1	0.001>
		** ACE-I and/or ARB**	10	13549/ 28660	46.89 (46.31, 47.47)	2098/ 4757	43.43 (42.02, 44.86)	1.49 (1.16, 1.92)	0.002	63.57	85.8	0.188
		**CombinationTherapy (e.g., Aspirin + Clopidogrel)**	2	104/ 595	17.19 (14.25, 20.35)	379/ 2161	16.98 (15.38, 18.64)	0.58 (0.12, 2.72)	0.489	9.94	89.9	0.001>
		**Diuretics**	3	1228/ 5613	21.78 (20.7, 22.87)	47/ 346	13.5 (10.05, 17.36)	1.67 (1.2, 2.31)	0.002	0.8	0	0.76
		**Insulin**	2	298/ 2858	10.24 (9.14, 11.4)	12/ 239	4.39 (2.05, 7.47)	2.62 (0.47, 14.51)	0.27	2.7	63	0.001>
**Treatment at Discharge**^a^	**Routine Drugs**	**Statin**	26	320427/ 385450	85.09 (84,98, 85.2)	26264/ 35197	76.07 (75.62, 76.52)	2.15 (1.23, 3.77)	0.007	7148.22	99.7	0.047
		**β-blockers**	26	168956/ 223566	77.36 (77.19, 77.54)	18672/ 28730	66.15 (65.6, 66.7)	2.16 (1.15, 4.07)	0.017	8143.30	99.7	0.282
		**CCB**	9	1228/ 16921	6.22 (5.84, 6.6)	394/ 1220	30.76 (28.17, 33.41)	0.37 (0.14, 1.07)	0.051	238.75	96.8	0.145
	**Anti-Platelet**	**Aspirin**	24	402008/ 487533	85.08 (84.98, 85.18)	23966/ 29355	83.2 (82.77, 83.63)	2.92 (1.54, 5.52)	0.001	6612.40	99.7	0.003
		**Clopidogrel**	9	75207/ 146941	51.49 (51.24, 51.75)	3204/ 8107	39.41 (38.34, 40.48)	5.2 (2.67, 10.15)	0.001>	733.08	98.9	0.06
		**P2Y12-Inhibitor**	9	230912/ 298482	78.39 (78.24, 78.53)	12901/ 18902	68.55 (67.88, 69.22)	3.53 (0.9, 13.91)	0.071	10092.57	99.9	0.22
		**Any-Anti-Platelet**	3	5404/ 7413	NA	176/ 524	32.61 (28.59, 36.75)	6.88 (3.84, 12.31)	0.001>	3.73	46.4	0.424
	**Anti-Coagulant**	**Anti-Coagulant**	2	50/ 567	8.71 (6.49, 11.21)	20/ 218	8.35 (4.96, 12.48)	1.23 (0.23, 6.51)	0.808	5.23	80.9	0.001>
		**LMWH**	3	7537/ 288310	1.57 (1.53, 1.62)	328/ 11900	2.41 (2.14, 2.7)	0.25 (0.01, 6.97)	0.411	185.97	98.9	0.879
	**ACE-I and/or ARB**	** RAAS-I (ACE-I + ARB)**	7	115612/ 172160	67.47 (67.24, 67.69)	9418/ 14442	65.58 (64.8, 66.35)	1.27 (0.3, 5.33)	0.744	6347.10	99.9	0.619
		**ACE-I**	5	130216/ 164438	79.46 (79.26, 79.66)	4703/ 6414	73.68 (72.57, 74.77)	1.75 (1.22, 2.5)	0.002	17.63	77.3	0.164
		**ARB**	2	60/ 941	5.6 (4.1, 7.28)	12/ 107	10.93 (5.42, 17.83)	0.56 (0.28, 1.12)	0.099	0.02	0	0.001>
		** ACE-I and/or ARB**	15	30688/ 49128	62.87 (62.44, 63.3)	5863/ 12005	48.88 (47.98, 49.77)	1.73 (1.47, 2.05)	0.001>	100.62	86.1	0.007
		**Nitrates**	7	20513/ 165411	12.02 (11.86, 12.18)	703/ 6658	9.45 (8.73, 10.2)	1.15 (0.6, 2.26)	0.693	76.84	92.2	0.743

**Variable**			Studies (n)	Pooled Prevalence of each Variable in Study Groups				**MINOCA **Vs **Control **(OR (95% CI))	*P*-value for OR=1	Cochrane Q	I^2^ (%)	*P*-value for Egger Test
				Sample Size/ Total **MINOCA**	Pooled Prevalenc in **MINOCA**	Sample Size/ Total **Control**	Pooled Prevalenc in **Control**					
**Treatment at Admission**^a^	**Routine Drugs**	**Statin**	5	3111/ 11684	25.65 (24.86, 26.45)	1876/ 8984	19.31 (18.48, 20.16)	3.48 (1.33, 9.1)	0.011	547.13	99.3	0.275
		**β-blockers**	5	3050/ 11684	25.95 (25.15, 26.75)	1772/ 8984	18.63 (17.81, 19.46)	2.34 (1.15, 4.73)	0.018	275.35	98.5	0.347
		**CCB**	NA	NA	NA	NA	NA	NA	NA	NA	NA	NA
	**Anti-Platelet**	** Aspirin**	2	1837/ 8964	20.49 (19.66, 21.33)	1270/ 5918	21.31 (20.27, 22.36)	1.07 (0.61, 1.89)	0.814	34.23	97.1	0.001>
		** Clopidogrel**	NA	NA	NA	NA	NA	NA	NA	NA	NA	NA
		**P2Y12-Inhibitor**	1	206/ 7266	2.84 (2.47, 3.24)	104/ 4069	2.56 (2.09, 3.09)	1.11 (0.88, 1.41)	0.382	NA	NA	NA
		**Any Anti-Platelet**	NA	NA	NA	NA	NA	NA	NA	NA	NA	NA
	**Anti-Coagulant**	** Warfarin**	NA	NA	NA	NA	NA	NA	NA	NA	NA	NA
		**Heparin**	NA	NA	NA	NA	NA	NA	NA	NA	NA	NA
		**LMWH**	NA	NA	NA	NA	NA	NA	NA	NA	NA	NA
		**Any-Anti-Coagulant**	1	375/ 7266	5.16 (4.66, 5.69)	90/ 4069	2.21 (1.78, 2.71)	2.41 (1.91, 3.04)	0.001>	NA	NA	NA
	**ACE-I and/or ARB**	**RAAS-I (ACE-I + ARB)**	NA	NA	NA	NA	NA	NA	NA	NA	NA	NA
		**ACE-I**	4	507/ 2350	21.22 (19.57, 22.92)	234/ 3247	5.21 (4.39, 6.09)	9.22 (1.4, 60.55)	0.021	107.58	97.2	0.449
		**ARB**	1	4/ 65	6.15 (1.7, 15.01)	0/ 12	0 (0, 26.46)	1.83 (0.09, 36.17)	0.692	NA	NA	NA
		**ACE-I and/or ARB**	2	1314/ 3830	33.51 (32.02, 35.01)	826/ 3556	21.97 (20.62, 23.34)	1.65 (1.41, 1.93)	0.001>	1.93	48.2	0.001>
		**CombinationTherapy (e.g., Aspirin + Clopidogrel)**	1	370/ 2132	17.35 (15.77, 19.03)	151/ 1707	NA	2.16 (1.77, 2.65)	0.001>	NA	NA	NA
		**Diuretics**	1	10/ 97	10.31 (5.06, 18.14)	8/ 98	8.16 (3.59, 15.45)	1.29 (0.49, 3.43)	0.605	NA	NA	NA
		**Insulin**	1	1/ 97	1.03 (0.03, 5.61)	2/ 98	2.04 (0.25, 7.18)	0.5 (0.04, 5.61)	0.574	NA	NA	NA
**Treatment at Discharge**^a^	**Routine Drugs**	**Statin**	10	14859/ 24654	61.17 (60.56, 61.79)	7778/ 23747	31.95 (31.35, 32.54)	2.42 (1.43, 4.1)	0.001	1171.03	99.2	0.678
		**β-blockers**	9	10921/ 19093	57.95 (57.24, 58.66)	5247/ 16683	NA	2.35 (1.15, 4.79)	0.019	1107.6	99.3	0.848
		**CCB**	2	25/ 139	17.6 (11.59, 24.5)	30/ 181	12.75 (8.2, 18.09)	2.19 (0.19, 25.26)	0.53	8.11	87.7	0.001>
	**Anti-Platelet**	**Aspirin**	7	8983/ 13182	70.8 (70, 71.58)	3759/ 11744	NA	1.85 (0.62, 5.5)	0.266	1262.27	99.5	0.819
		**Clopidogrel**	1	79/ 171	46.2 (38.56, 53.97)	30/ 180	16.67 (11.54, 22.93)	4.3 (2.62, 7.04)	0.001>	NA	NA	NA
		**P2Y12-Inhibitor**	3	5032/ 7311	69.33 (68.23, 70.41)	216/ 4319	4.15 (3.55, 4.79)	3.42 (0.16, 71.76)	0.428	47.96	95.8	0.289
		**Any-Anti-Platelet**	1	69/ 97	71.13 (61.05, 79.89)	4/ 98	4.08 (1.12, 10.12)	57.91 (19.42, 172.72)	0.001>	NA	NA	NA
	**Anti-Coagulant**	**Anti-Coagulant**	1	3.09 (0.64, 8.77)	3/ 97	2.04 (0.25, 7.18)	2/ 98	1.53 (0.25, 9.38)	0.644	NA	NA	NA
		**LMWH**	NA	NA	NA	NA	NA	NA	NA	NA	NA	NA
	**ACE-I and/or ARB**	** RAAS-I (ACE-I + ARB)**	1	41/ 97	61.86 (60.74, 62.97)	9/ 98	9.18 (4.29, 16.72)	7.24 (3.27, 16.04)	0.001>	NA	NA	NA
		** ACE-I**	NA	NA	NA	NA	NA	NA	NA	NA	NA	NA
		** ARB**	NA	NA	NA	NA	NA	NA	NA	NA	NA	NA
		** ACE-I and/or ARB**	4	4349/ 9553	45.49 (44.49, 46.5)	3362/ 10559	31.65 (30.76, 32.55)	2.03 (1.36, 3.03)	0.001	40.86	92.7	0.545
		**Nitrates**	2	1/ 120	0.4 (0, 3.12)	10/ 282	3.44 (1.53, 5.99)	0.48 (0.07, 3.03)	0.431	0.001>	0	0.001>

Compares the prescription rates of medications (e.g., statins, beta-blockers, ACE inhibitors) at admission and discharge among MINOCA, MIOCA, and control groups. The results highlight differences in treatment strategies across the groups, supported by statistical measures

*Abbreviations:* *NA* Not Available, *CCB* Calcium Channel Blocker, *LMWH* Low Molecular Weight Heparin, *RAAS-inhibitor* Renin-Angiotensin-Aldosterone System Inhibitor, *ACE-I* Angiotensin Converting Enzyme-Inhibitor, *ARB* Angiotensin Receptor Blockers

^a^Polled effect for qualitative values-was reported by OR

*P* values less than 0.05 were considered statistically significant

### Study characteristics

The characteristics of the studies are reported in Table 2. The studies included in this meta-analysis encompass a wide range of countries globally, such as China, Poland, New Zealand, Australia, the United Kingdom, the USA, Canada, Sweden, Germany, Italy, Denmark, Portugal, Slovenia, Brazil, Turkey, Israel, Spain, the Netherlands, Japan, and Egypt. This comprehensive analysis consisted of 112 studies comparing MINOCA with MIOCA (91 studies) and/or control groups (39 studies). A total of 5,908,768 patients were examined, including 2,504,298 patients in the MIOCA group, 739,727 patients in the MINOCA group, and 60,874 in the control group. Among patients undergoing coronary angiography, 8.92% (95% CI: 8.90–8.94) were diagnosed with MINOCA. The studies included in this meta-analysis featured various designs, including 83 cohort studies, 26 cross-sectional studies, and 3 case-control studies. The average follow-up period was 956.5 days. Additionally, the NOS (Newcastle-Ottawa Scale) used to assess study quality ranged from a score of 7 to 10. Of these, 5 studies scored 7, 21 studies scored 8, and 86 studies achieved a score of 9.

### Demographic and clinical characteristics of MINOCA

Table 3 presents of the baseline characteristics of the study population. Demographic analysis revealed that MINOCA patients were generally younger than MIOCA patients (61.52 ± 5.17 vs. 63.72 ± 5.62; SMD = 0.22) and more likely to be female (47.74% vs. 65.89%; OR = 2.36). Regarding medical history, MINOCA patients had a higher prevalence of obesity (44.34% vs. 38.71%; OR = 0.90), hypertension (72.81% vs. 71.76%; OR = 1.26), angina (20.15% vs. 15%; OR = 1.2), atrial fibrillation (11.07% vs. 5.22%; OR = 0.59), and chronic obstructive pulmonary disease (13.21% vs. 9.52%; OR = 0.67). However, they exhibited a lower prevalence of diabetes (26.49% vs. 30.65%; OR = 1.62), dyslipidemia (64.7% vs. 66.52%; OR = 1.54), and previous MI (16.22% vs. 24.09%; OR = 2.40) compared to MIOCA patients.

When compared to the control group, MINOCA patients were older (59.87 ± 4.94 vs. 55.05 ± 7.01; SMD = 0.4) and had a higher proportion of males (60.61% vs. 50.94%; OR = 1.25). In terms of medical history, MINOCA patients showed a higher prevalence of hypertension (59.45% vs. 43.9%; OR = 1.89), dyslipidemia (55.08% vs. 52.53%; OR = 1.97), obesity (39.17% vs. 36.65%; OR = 1.29), and diabetes (17.03% vs. 10.79%; OR = 1.52) compared to the general population.

### Clinical presentation, and laboratory data of MINOCA

Table 4 outlines the clinical presentation, and laboratory data of the study population. Atypical chest pain was notably higher in MINOCA patients (31.06% vs. 10.57%; OR = 0.58), while typical chest pain was more prevalent among MIOCA patients (24.79% vs. 14.83%; OR = 1.66). Additionally, when compared to the control group, MINOCA patients exhibited a slightly higher occurrence of typical chest pain (8.45% vs. 8.18%; OR = 0.87) and a significantly greater prevalence of dyspnea (7.76% vs. 5.47%; OR = 1.43). These findings suggest that the clinical symptoms of MINOCA and MIOCA patients differ in their manifestation, with MINOCA patients experiencing more atypical presentations. In addition, the results of laboratory data showed that the levels of C-reactive protein (CRP), creatinine kinase-myocardial infarction (CK-MB), Fibrinogen, Brain Natriuretic Peptide (BNP) and high-density lipoprotein (HDL) were significantly higher in MINOCA group compared to MIOCA (SMD= −0.01; 95%CI= −0.08, 0.1, SMD= −0.25; 95%CI=−0.16, 0.35, SMD= −0.42; 95%CI= −0.26, 0.58, SMD= −0.42; 95%CI= −0.14, 0.71, SMD= −0.25 ; 95%CI= −0.38, −0.12, respectively). while low-density lipoprotein (LDL) (SMD = 0.18; 95%CI = 0.36, 0.002), and total cholesterol (TC) (SMD = 0.17; 95%0.37, 0.02) levels were significantly lower.

### Medication prescription in MINOCA patients

Table 5 shows the prescribed drugs. The analysis of medication prescription patterns in MINOCA patients was conducted at two stages: during hospitalization and at discharge. The findings revealed that the most commonly prescribed drugs during hospitalization were aspirin (71.91%), anti-platelet agents (62.68%), and β-blockers (52.65%). At discharge, the most frequently prescribed medications included aspirin (83.2%), statins (76.07%), and ACE-I (73.68%).

When compared to the MIOCA group, MINOCA patients received a higher rate of anticoagulant medications during hospitalization, such as Warfarin (7.5% vs. 3.25%; OR = 0.41) and other anticoagulants (5.2% vs. 3.02%; OR = 0.56). At discharge, the use of anticoagulants (LMWH: 2.41% vs. 1.57%; OR = 0.25) and angiotensin receptor blockers (ARB) (10.93% vs. 5.6%; OR = 0.56) was also more common in MINOCA patients compared to those with MIOCA.

### Prognosis of MINOCA patients

The prognostic factors of MINOCA patients are shown in Table 6. To assess the prognosis of MINOCA, both short-term and long-term outcomes were analyzed. The most frequent short-term outcomes observed in MINOCA patients were revascularization (11.11%) and major adverse cardiac events (MACE) (5.34%). In the long term, the most common outcomes included revascularization through percutaneous coronary intervention (Re-PCI) (5.64%), coronary artery bypass grafting (CABG) (4.05%), and cardiovascular death (CV-Death) (2.23%).

**Table 6:** Comparison of in-hospital and one-year follow-up outcomesin MINOCA, MIOCA and control

**Variable**		Studies (n)	Pooled Prevalence of each Variable in Study Groups				**MIOCA **Vs **MINOCA** (RR (95% CI))	*P*-value for RR=1	Cochrane Q	I^2^ (%)	*P*-value for Egger Test
			Sample Size/ Total **MIOCA**	Pooled Prevalenc in **MIOCA **(%)	Sample Size/ Total** MINOCA**	Pooled Prevalenc in **MINOCA **(%)					
**Short-Term Outcomes (In-Hospital Outcomes)**^**a**^	**CV-Death**	5	1649/ 64030	2.51 (2.39, 2.63)	37/ 4402	0.68 (0.44, 0.97)	2.7 (1.79, 4.1)	0.001>	5.94	32.6	0.179
	**MACE**	6	31912/ 288769	10.91 (10.8, 11.03)	1171/ 20063	5.34 (5.03, 5.66)	3.15 (1.82, 5.47)	0.001>	50.90	90.2	0.134
	**Cardia Arrest**	1	3089/ 160886	1.92 (1.85, 1.99)	55/ 6063	0.91 (0.68, 1.18)	2.12 (1.62, 2.76)	0.001>	NA	NA	NA
	**MI**	7	11119/ 1456110	0.72 (0.71, 0.73)	107/ 245941	0 (0, 0)	7.41 (3.12, 17.57)	0.001>	24.81	75.8	0.073
	**CVA**	5	3906/ 1271503	0.25 (0.24, 0.26)	207/ 238191	0 (0, 0)	1.33 (0.49, 3.59)	0.576	24.15	83.4	0.07
	**Stroke**	3	8346/ 435056	1.38 (1.35, 1.42)	302/ 23210	1 (0.87, 1.14)	1.26 (0.56, 2.85)	0.577	10.24	80.5	0.805
	**HF**	1	42/ 4239	0.99 (0.71, 1.34)	2/ 298	0.67 (0.08, 2.4)	1.48 (0.36, 6.07)	0.589	NA	NA	NA
	**Cardiogenic Shock**	6	8243/ 1431435	0.44 (0.43, 45)	156/ 244166	0 (0, 0)	4.1 (1.61, 10.33)	0.003	72.34	93.1	0.410
	**Tamponade**	3	3558/ 273043	1.25 (1.2, 1.29)	45/ 17108	0.07 (0.02, 0.14)	4.96 (3.7, 6.66)	0.001>	1.57	0	0.913
	**Pulmonary Edema**	3	1130/ 164059	0.59 (0.55, 0.63)	24/ 6314	0.1 (0.02, 0.24)	1.84 (0.76, 4.47)	0.179	6.99	71.4	0.785
	**Pericarditis**	1	25/ 2761	0.91 (0.59, 1.33)	0/ 142	0 (0, 2.56)	2.64 (0.16, 43.15)	0.496	NA	NA	NA
	**Bleeding**	9	27623/ 1562233	1.61 (1.59, 1.63)	1295/ 257081	0.34 (0.32, 0.37)	1.6 (0.82, 3.1)	0.171	170.33	95.3	0.07
	**Revascularization**	1	44454/ 269931	16.47 (16.33, 16.61)	1872/ 16849	11.11 (10.64, 11.59)	1.48 (1.42, 1.55)	0.001>	NA	NA	NA
	** -PCI**	6	20903/ 38834	54.49 (54, 54.99)	150/ 10189	0.94 (0.74, 1.15)	2.7 (1.54, 5.52)	0.001>	139.95	96.4	0.179
	** -CABG**	7	7899/ 49091	14.15 (13.84, 14.46)	126/ 10601	0.23 (0.13, 0.35)	9.15 (1.31, 63.81)	0.025	585.55	99	0.212
**Long-Term Outcomes (One-Year Follow-up)**^a^	**Death**	18	27109/ 291233	8.97 (8.87, 9.08)	1221/ 17099	6.34 (5.98, 6.72)	1.48 (1.17, 1.86)	0.001	134.81	87.4	0.103
	**CV-Death**	10	2749/ 66155	4.01 (3.86, 4.16)	92/ 5855	1.26 (0.96, 1.59)	2.23 (1.67, 2.98)	0.001>	12.21	26.3	0.979
	**MACE**	8	3967/ 22160	16.49 (16, 16.98)	185/ 3449	4.84 (4.12, 5.61)	2.13 (1.45, 3.14)	0.001>	33.56	79.1	0.488
	**MI**	11	15363/ 233419	6.28 (6.18, 6.38)	358/ 12856	2.24 (1.98, 2.51)	2.3 (1.79, 5.01)	0.001>	102.48	90.2	0.367
	**CVA**	4	173/ 11849	1.04 (0.86, 1.24)	4/ 836	0.08 (0, 0.52)	2.05 (0.88, 4.77)	0.098	0.7	0	0.619
	**Stroke**	8	3672/ 33609	7.16 (6.88, 7.43)	195/ 5029	186 (1.48, 2.27)	1.58 (1.25, 1.99)	0.001>	9.42	25.7	0.182
	**HF**	4	547/ 35987	1.36 (1.24, 1.48)	68/ 4372	1.36 (1.01, 1.74)	1.17 (0.91, 1.51)	0.217	0.39	0	0.927
	**Re-Bleeding**	3	1127/ 9026	11.77 (11.11, 12.44)	27/ 835	3.02 (1.92, 4.33)	1.99 (0.99, 3.99)	0.051	4.19	52.3	0.056
	**Revascularization**	2	174/ 2492	6.77 (5.81, 7.8)	14/ 240	5.02 (2.52, 8.24)	0.98 (0.05, 19.75)	0.99	13.54	92.6	0.001>
	**Re-PCI**	4	33919/ 15693		270/ 2704		5.64 (0.25, 127.86)	0.277	833.56	99.6	0.414
	**Re-CABG**	5	10156/ 177395	5.58 (5.47, 5.69)	199/ 8718	1.73 (1.45, 2.04)	4.05 (1.17, 13.97)	0.027	35.18	88.6	0.265

**Variable**		Studies (n)	Pooled Prevalence of each Variable in Study Groups				**MINOCA**Vs **Control**(RR (95% CI))	*P*-value for ROR=1	Cochrane Q	I^2^ (%)	*P*-value for Egger Test
			Sample Size/ Total **MINOCA**	Pooled Prevalenc in **MINOCA**	Sample Size/ Total **Control**	Pooled Prevalenc in **Control**					
**Short-Term Outcomes (In-Hospital Outcomes)**^a^	**CV-Death**	NA	NA	NA	NA	NA	NA	NA	NA	NA	0.082
	**MACE**	1	1/ 115	NA	2/ 266	0.75 (0.09, 2.69)	1.16 (0.11, 12.63)	0.905	NA	NA	NA
	**Cardia Arrest**	NA	NA	NA	NA	NA	NA	NA	NA	NA	NA
	**MI**	NA	NA	NA	NA	NA	NA	NA	NA	NA	NA
	**CVA**	NA	NA	NA	NA	NA	NA	NA	NA	NA	NA
	**Stroke**	NA	NA	NA	NA	NA	NA	NA	NA	NA	NA
	**HF**	1	2/ 298	0.67 (0.08, 2.4)	0/ 256	0 (0, 1.43)	4.3 (0.21, 89.11)	0.346	NA	NA	NA
	**Cardiogenic Shock**	1	2/ 298	0.67 (0.08, 2.4)	1/ 256	NA	1.72 (0.16, 18.83)	0.658	NA	NA	NA
	**Tamponade**	NA	NA	NA	NA	NA	NA	NA	NA	NA	NA
	**Pulmonary Edema**	NA	NA	NA	NA	NA	NA	NA	NA	NA	NA
	**Pericarditis**	NA	NA	NA	NA	NA	NA	NA	NA	NA	NA
	**Bleeding**	1	2/ 298	0.67 (0.08, 2.4)	1/256	0.39 (0.01, 2.16)	1.72 (0.16, 18.83)	0.658	NA	NA	NA
	**Revascularization**	NA	NA	NA	NA	NA	NA	NA	NA	NA	NA
	** -PCI**	1	82/ 8384	0.98 (0.78, 1.21)	15/ 8391	0.18 (0.1, 0.29)	5.47 (3.16, 9.48)	0.001>	NA	NA	NA
	** -CABG**	1	11/ 8384	0.13 (0.07, 0.23)	2/ 8391	0.02 (0, 0.09)	5.51	0.026	NA	NA	NA
**Long-Term Outcomes (One-Year Follow-up)**^a^	**Death**	3	82/ 2167	3.53 (2.79, 4.36)	6/ 1525	0.33 (0.07, 0.74)	5.2 (1.27, 20.97)	0.022	3.24	38.3	0.086
	**CV-Death**	2	33/ 1996	1.57 (1.05, 2.19)	2/ 1345	0.11 (0, 0.41)	8.13 (1.37, 48.27)	0.021	1.33	25.1	0.001>
	**MACE**	1	2/ 312	0.64 (0.08, 2.3)	2/ 545	0.37 (0.04, 1.32)	1.75 (0.25, 12.34)	0.576	NA	NA	NA
	**MI**	1	1/229	0.44 (0.01, 2.41)	2/ 196	NA	0.43 (0.04, 4.68)	0.487	NA	NA	NA
	**CVA**	NA	NA	NA	NA	NA	NA	NA	NA	NA	NA
	**Stroke**	2	77/ 1996	3.69 (2.89, 4.58)	8/ 1345	0.57 (0.21, 1.07)	4.14 (0.91, 18.72)	0.065	2.24	55.4	0.001>
	**HF**	1	31/ 1774	1.75 (1.19, 2.47)	2/1027	0.19 (0.02, 0.7)	8.97 (2.15, 37.42)	0.003	NA	NA	NA
	**Re-Bleeding**	NA	NA	NA	NA	NA	NA	NA	NA	NA	NA
	**Revascularization**	NA	NA	NA	NA	NA	NA	NA	NA	NA	NA
	**Re-PCI**	NA	NA	NA	NA	NA	NA	NA	NA	NA	NA
	**Re-CABG**	NA	NA	NA	NA	NA	NA	NA	NA	NA	NA

Compares differences in short-term (in-hospital) and long-term (one-year follow-up) outcomes, such as mortality, myocardial infarction, and heart failure rates, across MINOCA, MIOCA, and control groups. Statistical analyses emphasize variations in prognosis among the groups

*Abbreviations:* *MACE* Major Adverse Cardiac Events, *CV-Death* Cardio-Vascular Death, *MI* Myocardial Infarction, *CVA* Cerebro-Vascular Accident, *HF* Heart Failure, *PCI* Percutaneous Coronary Intervention, *CABG* Coronary Artery Bypass Graft

^a^Polled effect for qualitative values-was reported by OR

*P* values less than 0.05 were considered statistically significant

Compared to the MIOCA group, MINOCA patients showed a significantly lower prevalence of both short-term and long-term adverse outcomes. Percutaneous coronary intervention (PCI) was notably more common as a short-term outcome in MIOCA patients (54.49% vs. 0.94%; OR = 2.7). Similarly, MACE was more prevalent as a long-term outcome in MIOCA patients (16.49% vs. 4.84%; OR = 2.13).

Interestingly, certain short-term outcomes, including MI, cerebrovascular accidents (CVA), cardiogenic shock, and pericarditis, were entirely absent in the MINOCA group, with a prevalence of 0%.

## Discussion

### Main findings

Due to the distinctive characteristics of MINOCA, it poses substantial risks to cardiac health [94]. Moreover, in many cases, it is recognized as a diagnostic challenge [81]. To our knowledge, this meta-analysis represents the most comprehensive examination of MINOCA compared to MIOCA and controls to date. The results provide critical insights into the demographic, clinical, and laboratory features, as well as the prognosis and treatment of MINOCA patients. Among patients undergoing coronary angiography, the pooled prevalence of MINOCA was 8.92% (95% CI: 8.90–8.94), reaffirming its clinical significance as a distinct condition.

Demographic analysis revealed significant differences between MINOCA and MIOCA patients. MINOCA patients were generally younger (mean age 61.52 vs. 63.72 years) and predominantly female (65.89% vs. 47.74%), reflecting potential hormonal influences on disease mechanisms. For instance, estrogen’s protective cardiovascular effects diminish after menopause, possibly explaining the higher prevalence of MINOCA in postmenopausal women [3, 49, 75, 85]. Additionally, MINOCA patients showed higher rates of obesity, hypertension, atrial fibrillation, and chronic obstructive pulmonary disease, whereas MIOCA patients exhibited greater prevalence of diabetes, dyslipidemia, and prior myocardial infarction. Obesity and hypertension may contribute to coronary microvascular dysfunction and inflammatory processes without causing significant obstruction [35, 131]. By contrast, diabetes and dyslipidemia, which promote macrovascular atherosclerosis, are less prominent in MINOCA [61]. These findings highlight the key differences in the risk factors between MINOCA and MIOCA. Therefore, a deeper understanding of the molecular pathways involved in MINOCA can lead to the development of new diagnostic and therapeutic approaches for better management of these patients.

This meta-analysis revealed that MIOCA patients experience significantly higher rates of typical chest pain, whereas atypical chest pain is more prevalent among MINOCA patients. Additionally, dyspnea occurred more frequently in MINOCA patients compared to MIOCA patients (7.76% vs. 5.47%). Laboratory findings demonstrated that MINOCA patients had elevated levels of CK-MB, CRP, BNP, fibrinogen, and HDL compared to MIOCA patients. These findings, when considered alongside the clinical symptoms of MINOCA, become particularly important. Elevated CRP and fibrinogen levels indicates systemic inflammation and immune activation, which leads to vascular spasms and microcirculatory dysfunction [36]. This mechanism can explain the occurrence of atypical chest pain in MINOCA patients. CRP and fibrinogen contribute to inflammation by activating pathways such as NF-κB, leading to increased production of pro-inflammatory cytokines like IL-6 and TNF-α, which may trigger atypical cardiac symptoms [36, 39]. Furthermore, microcirculatory failure prompts the heart to increase intraventricular pressure, resulting in higher BNP production to regulate blood pressure and volume [36]. Elevated BNP levels are associated with a higher incidence of dyspnea in MINOCA patients, as ischemia-induced fluid buildup in the lungs can cause shortness of breath [42, 110]. Additionally, lipid profiles showed significantly lower LDL and TC levels in MINOCA patients compared to MIOCA patients, suggesting that vascular obstruction and atherosclerosis play a lesser role in MINOCA [66, 87, 96]. Instead, functional microcirculatory disorders and vascular spasms appear to be the primary mechanisms driving MINOCA. Reduced LDL levels in MINOCA patients might be attributed to heightened antioxidant activity or abnormalities in lipolytic pathways [31, 106]. Overall, these findings highlight significant differences in clinical symptoms and laboratory parameters between MINOCA and MIOCA patients. These differences provide valuable insights for clinicians and researchers, emphasizing the need for a deeper understanding of the molecular pathways underlying MINOCA to inform more effective diagnostic, therapeutic, and management strategies.

In this study, the patterns of medication prescription were analyzed during two critical stages: the hospitalization period and at discharge. the primary goal is to control clinical symptoms and prevent the progression of cardiac damage [53]. Medications such as aspirin (to prevent microthrombosis formation), antiplatelet agents (to enhance aspirin’s effect), β-blockers (to control heart rate), and anticoagulants (to prevent the formation of small clots) are commonly prescribed [11, 49, 53, 72]. At discharge, the focus shifts to long-term disease management and reducing the risk of recurrence [60, 78]. Aspirin, statins (to reduce inflammation and prevent atherosclerotic plaque formation), ACE-I (to improve cardiac function), ARBs (to control blood pressure), and anticoagulants (to minimize microthrombosis) are frequently prescribed [10, 81]. These findings indicate that medication patterns in MINOCA patients are stage-specific, aligning with their clinical condition to improve outcomes and reduce future risks.

Compared to MIOCA patients, MINOCA patients experienced significantly less severe short- and long-term adverse outcomes. For instance, PCI as a short-term outcome was notably more common in MIOCA patients (54.49% vs. 0.94%), while long-term MACE were reported with higher prevalence in MIOCA patients (16.49% vs. 4.84%). This disparity is likely due to the absence of significant mechanical vascular obstructions in MINOCA, reducing the need for invasive interventions, while MIOCA patients face higher risks due to extensive vascular blockages [29, 44]. Additionally, in MINOCA patients, vascular spasms and inflammation rarely result in acute heart failure or large vessel obstructions, which may explain the absence of outcomes like MI, cardiogenic shock, and CVA observed in this study [61, 81, 90]. Furthermore, CV-Death was significantly lower in MINOCA patients compared to MIOCA patients. Symptoms like dyspnea in MINOCA patients may stem from vasomotor dysfunction and increased intraventricular pressure rather than severe vascular obstructions. This mechanism, linked to elevated BNP secretion, can cause heart failure symptoms without leading to serious complications like CV-Death [33, 37, 112]. These findings underscore the better overall prognosis for MINOCA patients, emphasizing the functional rather than structural nature of the disease and highlighting the need for targeted management strategies to prevent future complications [38].

The pathophysiology of MINOCA is multifactorial, involving mechanisms such as coronary artery spasm, microvascular dysfunction, and plaque disruption [13, 132]. Coronary artery spasm, often identified through acetylcholine provocation testing, can cause transient myocardial ischemia despite non-obstructive coronary arteries [133]. Microvascular dysfunction, common in women with MINOCA, is characterized by impaired coronary flow and can be assessed using tools like coronary flow reserve (CFR) or index of microvascular resistance (IMR) [104, 134]. Plaque disruption, including rupture or erosion, may also contribute, with advanced imaging techniques like IVUS and OCT revealing subtle atherosclerotic changes undetectable on conventional angiography [135]. These mechanisms highlight the need for personalized treatment strategies. For instance, vasospastic or microvascular dysfunction may benefit from calcium channel blockers or Aspirin, while patients with plaque disruption may respond better to antiplatelet or anticoagulant therapy. Future research should focus on stratifying MINOCA patients by underlying mechanisms to optimize diagnostic and therapeutic approaches.

Although the underlying mechanisms of MINOCA remain incompletely understood, the reduced prescription rates of critical medications such as β-blockers, ACE-Is, statins, and antiplatelet may partially account for unfavorable outcomes in some cases [136, 137]. Our findings emphasize the importance of optimizing medical therapy for MINOCA patients, even in the absence of obstructive coronary artery disease. Clinicians should adopt a more comprehensive approach to prescribing these medications in patients with ACD symptoms to improve outcomes and address residual cardiovascular risks. Tailoring treatment protocols based on individual patient characteristics and advancing research into the specific mechanisms underlying MINOCA are critical steps for developing effective, evidence-based management strategies for this unique patient population.

### Strengths and limitations

This meta-analysis holds significant implications for several reasons. First, the inclusion of numerous studies with large sample sizes ensures robust and consistent results. Second, we conducted comparisons between MINOCA and two different groups (MIOCA and control), providing comprehensive insights into various aspects of these patients. Third, almost all aspects of these patients (clinical characteristics, laboratory data, vital signs, echocardiography, and ECG results at admission time, symptoms at arrival to the hospital, medication use (at admission to the hospital and discharge), and short-term (in-hospital) and long-term (one-year follow-up) prognosis was investigated relative to both MIOCA and control group.

However, it is important to consider some limitations in our study. First, given the complexity and variability of MINOCA, it is important to examine the heterogeneity observed in the pooled results. The observed heterogeneity (I² > 99%) likely reflects differences in study designs, patient populations, and methodological approaches. For instance, some studies focused on specific patient subgroups, such as those with heart failure [85] or COVID-19 [112], while others excluded patients with prior coronary artery disease [81, 138] or included those with chronic stable angina [6, 139]. While we standardized the definition of MINOCA in our inclusion criteria according to the ESC guidelines [140], differences in study methodologies and patient characteristics could still influence the results. Second, most studies included were retrospective, which might limit the external validity of the findings. However, the large sample size and consistent findings across studies help mitigate this limitation. Third, Patients who did not undergo angiography were excluded, potentially introducing selection bias. This exclusion limits the applicability of our results to patients without angiographic evaluation and may overlook potential cases of MINOCA diagnosed through advanced imaging techniques such as cardiac MRI or CT coronary angiography. Future research should include non-angiographic populations to enhance the generalizability of findings. Fourth, the underlying mechanisms of MINOCA, which may vary considerably among patients, could not be established from the data available in this large registry. Understanding these mechanisms is crucial for tailored treatment, and future studies should focus on elucidating these pathways. Fifth, Interactions between discharge medical therapy and adverse cardiovascular events at long-term follow-up could not be explored. This limitation underscores the need for more prospective studies to assess the long-term impact of various treatments on MINOCA outcomes. Finally, potential residual confounding factors, such as unmeasured comorbidities or variations in healthcare settings, may have influenced the outcomes, highlighting the importance of controlled prospective designs in future studies.

## Conclusion

This meta-analysis highlights critical differences between MINOCA and MIOCA, underscoring the unique clinical characteristics, laboratory findings, and outcomes of MINOCA patients. Key findings indicate that MINOCA primarily affects younger, predominantly female patients and is characterized by atypical chest pain, dyspnea, and elevated inflammatory markers such as CRP, BNP, and fibrinogen. These observations suggest that microvascular dysfunction and vascular spasm are central to the pathophysiology of MINOCA, contrasting with the macrovascular atherosclerosis predominant in MIOCA. The prognosis for MINOCA patients is notably better, with significantly lower rates of short- and long-term adverse cardiovascular events, including MACE and cardiovascular death. These findings emphasize the importance of optimizing management strategies tailored to MINOCA’s unique mechanisms, including targeted anti-inflammatory therapies and medications that address microvascular dysfunction. To improve outcomes further, future research should prioritize developing standardized diagnostic criteria and exploring novel therapeutic approaches specific to MINOCA. These efforts can enhance patient care and bridge existing gaps in the understanding and treatment of this distinct clinical entity.

## Data Availability

The present study data are available from the corresponding author upon reasonable request.
